# Herpes simplex virus 1 accelerates the progression of Alzheimer’s disease by modulating microglial phagocytosis and activating NLRP3 pathway

**DOI:** 10.1186/s12974-024-03166-9

**Published:** 2024-07-18

**Authors:** Zhimeng Wang, Jing Liu, Jing Han, Tianyi Zhang, Shangjin Li, Yanfei Hou, Huili Su, Fangping Han, Conggang Zhang

**Affiliations:** 1 School of Pharmaceutical Sciences, Tsinghua University, Beijing, 100084 China; 2 State Key Laboratory of Membrane Biology, Tsinghua-Peking Center for Life Sciences, Beijing Frontier Research Center of Biological Structure, SXMU-Tsinghua Collaborative Innovation Center for Frontier Medicine, Tsinghua University, Beijing, 100084 China; 3 State Key Laboratory of Stem Cell and Reproductive Biology, Institute of Zoology, Chinese Academy of Sciences, Beijing, 100101 China; 4 School of Pharmaceutical Sciences, IDG/McGovern Institute for Brain Research, Tsinghua-Peking Joint Center for Life Sciences, Tsinghua University, Beijing, 100084 China

## Abstract

Accumulating evidence implicates that herpes simplex virus type 1 (HSV-1) has been linked to the development and progression of Alzheimer’s disease (AD). HSV-1 infection induces β-amyloid (Aβ) deposition in vitro and in vivo, but the effect and precise mechanism remain elusive. Here, we show that HSV-1 infection of the brains of transgenic 5xFAD mice resulted in accelerated Aβ deposition, gliosis, and cognitive dysfunction. We demonstrate that HSV-1 infection induced the recruitment of microglia to the viral core to trigger microglial phagocytosis of HSV-GFP-positive neuronal cells. In addition, we reveal that the NLRP3 inflammasome pathway induced by HSV-1 infection played a crucial role in Aβ deposition and the progression of AD caused by HSV-1 infection. Blockade of the NLRP3 inflammasome signaling reduces Aβ deposition and alleviates cognitive decline in 5xFAD mice after HSV-1 infection. Our findings support the notion that HSV-1 infection is a key factor in the etiology of AD, demonstrating that NLRP3 inflammasome activation functions in the interface of HSV-1 infection and Aβ deposition in AD.

**Supplementary Information:**

The online version contains supplementary material available at 10.1186/s12974-024-03166-9.

## Introduction

Alzheimer’s disease (AD) is the most common neurodegenerative disorder, resulting in progressive memory loss and dementia. It exists in two forms: a genetic etiology of early-onset familial AD (fAD) caused by mutations of genes, such as *APP*, *PSEN1* and *PSEN2* genes; and a sporadic form of late-onset AD (LOAD) caused by a complex multifactorial etiology [1–3]. The deposition of senile plaques enriched with β-amyloid (Aβ) peptides is a pathological hallmark of both fAD and LOAD [4]. Aβ is primarily produced in neurons through the sequential processing of APP by β-secretase/BACE1 and γ-secretase/Presenilin [5, 6]. Traditionally, Aβ has been identified as the primary cause of the disease in fAD, known as the “Aβ cascade hypothesis”, where increased production of toxic Aβ species initiates a series of progressive changes that ultimately lead to neurodegeneration [7, 8]. However, the precise mechanism of Aβ production and clearance in LOAD is not clear.

Mounting evidence suggests that pathogens are involved in the progression of AD pathogenesis [9–11]. The AD pathogen hypothesis suggests that viral or microbial infection of the central nervous system (CNS) may act as triggers to induce a pathological cascade, leading to accumulation of Aβ [12–14]. Several pathogens, including *Chlamydophila pneumoniae* [15], *Borrelia spirochetes* [16], *Candida glabrata* [17], human herpesviruses 6 and 7 (HHV6 and HHV7) [13] and herpes simplex virus 1 (HSV-1) [11, 18, 19], have been linked to LOAD. Among these pathogens, HSV-1 has emerged as one of the leading pathogens and has been proposed as a potential risk factor in AD pathogenesis [20, 21]. Independent cohort studies have revealed that HSV-1 is strongly associated with neurodegenerative diseases, especially AD [20, 22]. It has been established that HSV-1 is present in the brains of a high proportion of elderly normal subjects and Alzheimer’s disease (AD) patients [23, 24].

HSV-1 is a neurotropic double-strand DNA virus that establishes a latent infection in the sensory neurons of trigeminal ganglia of humans [25]. HSV-1 brain infection leads to the activation of glial cells, especially microglia cells [26]. Microglia cells, the resident innate immune cells in the central nervous system, play essential roles in healthy and neurodegenerative diseases [27]. In physiological conditions, resting microglia have ramified processes that constantly survey the microenvironment, assisting neuronal development, synaptic pruning and support neuronal survival [28, 29]. In response to pathogen infections or neurodegenerative disorders, reactive microglia mediate phagocytic uptake and the secretion of inflammatory cytokines and chemokines [30–32]. HSV-1 infection activates the cGAS-STING signaling pathway and NLRP3 inflammasome pathway in microglia to fight against viral invsion [33–36]. In the other hands, the pathogen-mediated innate immune response also could cause neuroinflammation, which is an important hallmark of neurodegenerative diseases. However, the mechanism of microglia activation and innate immune signaling in AD pathology upon HSV-1 infection remains largely unclear. Therefore, a comprehensive study of microglia, Aβ deposition, and neuroinflammation after HSV-1 infection might deepen our understanding of AD.

In this study, we administrated HSV-1-GFP into 5xFAD mice and investigated the effects of HSV-1 infection on Aβ deposition and cognitive function. The results revealed that HSV-1 infection led to Aβ deposition and cognitive deficits in 5xFAD mice. Following HSV-1 infection, microglia were recruited to the viral core but not Aβ deposition, enhancing their phagocytic uptake of viruses, thereby leading to the accumulation and deposition of Aβ. Moreover, the NLRP3 inflammasome signaling was activated after HSV-1 infection and drove the accumulation of Aβ aggregates. Administration of MCC950 sodium, a selective small-molecular inhibitor of the NLRP3 inflammasome, reduced Aβ deposition and ameliorated cognitive decline in 5xFAD mice following HSV-1 infection. Together, our findings support the concept that HSV-1 infection and NLRP3 inflammasome pathway are connected to the development and progression of AD pathology.

## Materials and methods

### Transgenic mice and mouse infection model

The 5xFAD transgenic mice (B6SJL-Tg (APPSwFlLon, PSEN1*M146L*L286V) 6799Vas/Mmjax, 34840-JAX) were originally obtained from the Jackson laboratory and maintained on the C57BL/6 background. For behavioral tests, 3-month-old male 5xFAD mice were used. For other in vivo experiments, age and sex-matched mice were used. Mice were housed with free access to water and food under a 12 h/12 h light–dark cycle. All experimental animal procedures were approved by the Institutional Animal Care and Use Committee of Tsinghua University.

For behavioral tests, 3-month-old male 5xFAD mice received bilateral injections of HSV-1-GFP viron suspension at coordinates AP, − 2.0; ML, ± 1.5; DV, − 2.0. This injection was carried out using a 5 μL syringe equipped with a 30-gauge needle attached to a digital stereotaxic apparatus and an infusion pump, administrated at a rate of 0.2 μL/min. Following the completion of each injection, the needle was retained in place for 5 min before gradual withdrawal. 21 days after the injections, the mice were subjected to a battery of behavioral tests.

In the case of other in vivo experiments, we employed the approach involved by Eimer and coauthors [13]. In brief, age and sex-matched 5xFAD mice received unilateral injections of HSV-1-GFP viron suspension at the coordinates AP, − 2.0; ML, − 1.5; DV, − 2.0 (right side) using a 5 μL syringe fitted with a 30-gauge needle attached to a digital stereotaxic apparatus and an infusion pump, administrated at a rate of 0.2 μL/min. Sterile PBS was injected into the contralateral side of the brain as controls (left side). Subsequently, the mice were either perfused for immunostaining or euthanized for western blotting at the indicated time points.

### Cell culture

BV2 cells, HT22 cells and Vero cells were cultured in Dulbecco’s modified Eagle medium (DMEM) (Gibco) supplemented with 10% (v/v) fetal bovine serum (FBS) (Gemini), 100 U/ml penicillin and 100 μg/ml streptomycin. All these cells are grown at 37 °C in a humidified atmosphere containing 5% CO_2_.

Primary microglial cells were derived from P0-P5 WT mice as previously described [4, 37]. Briefly, neonatal cortex was collected, trypsinized for 10 min, and filtered through a 70 μm filter. Cells were then cultured in DMEM medium containing 10% FBS and 1% penicillin/streptomycin for 2–3 weeks. The medium was replaced with fresh cell culture medium every three days. The microglia were isolated by shaking (200–220 rpm, 3 h) after 2–3 weeks of primary cultivation. The medium containing detached microglia was collected and seeded into PDL-coated 12-well or 24-well plates. After the cells attached, the medium was replaced with fresh cell culture medium.

### Virus production

Herpes simplex virus 1-GFP (HSV-1-GFP, KOS Strain, generously provided by Dr. Daxing Gao, University of Science and Technology of China), was employed in the innate immune studies [38]. The virus was amplified in Vero cells as described in previous paper [39, 40]. The supernatant containing the virus was harvested and centrifuged at 5000 rpm/4 °C. The viral suspension was filtered through a 0.22 μm filter and purified by ultracentrifugation as 20,000 rpm/2 h. The concentrated virus was aliquoted and stored at − 80 °C.

### Immunofluorescence

Mice were anesthetized with 5% chloral hydrate and perfused with cold PBS, followed by 4% paraformaldehyde (Aladdin, PH 7.4). Subsequently, mouse brains were harvested and coronally sectioned into 40 μm-thick serial sections. The brain sections were then washed three times with PBS and blocked for 2 h with a blocking buffer (3% BSA containing 0.1% Triton X-100) at room temperature, followed by overnight incubation with primary antibodies against MOAB2 (1:1000, Cat # ab126649, Abcam), Iba1 (1:1000, Ca t# 019-19741, Wako, PRID: AB_839504), GFAP (1:1000, Cat # 13-0300, Invitrogen, PRID: AB_86543), CD3 (1:1000, Cat #ab135372, PRID: AB_2884903), F4/80 (1:500, Cat #ab300421, PRID: AB_2936298), ASC (1:500, Cat #AG-25B-0006, Adipogen, PRID: AB_2490440), CD68 (1:1000, Cat #ab53444, Abcam, PRID: AB_869007), NLRP3 (1:1000, Cat #AG-20B-0014, Adipogen, PRID: AB_2490202) at 4 °C. After washing, the sections were labeled with fluorescent secondary antibodies conjugated to Alexa Fluor 568/594/647 in blocking buffer containing DAPI (Invitrogen). The slides were then observed with a fluorescence microscopy (ZEISS LSM780 microscopy or OLYMPUS FV3000RS-BX microscopy).

For the Aβ and HSV-GFP phagocytosis in vitro assay, HT22 cells were infected with HSV-1-GFP for 24 h, then washed and suspended with PBS. BV2 and primary microglial cells were incubated with 1 μg/ml Alexa 555-labeled Aβ_1-42_ (Anaspec), or 1 μg/ml Alexa 555-labeled Aβ_1-42_ plus HSV-1-GFP from HT-22 neuronal cells for 4 h. Subsequently, cells were fixed with 4% paraformaldehyde for 20 min at room temperature and stained with DAPI. The cells were then imaged with the Zeiss LSM780.

### Thioflavin S staining

Thioflavin S staining was used to label the Aβ plaques. Briefly, brain sections were stained with 0.1% Thioflavin S (Thio-S, sigma) in the dark for 8 min in 50% ethanol, followed by two washes with 50% ethanol and three washes with PBS. Subsequently, the sections were mounted for imaging.

### Microscopy and image analysis

All imaging was performed using an OLYMPUS FV3000RS-BX and ZEISS 780 Confocal laser scanning microscope with × 4, × 10, × 20, × 40 and × 63 objectives. For Aβ plaques quantification, ten views from the hippocampus of five mice and the entire brain were assessed. For microglia, astrocyte and inflammasome markers quantification, Iba1-, GFAP-, CD68-, ASC-, and NLRP3-positive cells were counted and then calculated as the Iba1^+^, GFAP^+^, CD68^+^, ASC^+^ and NLRP3^+^ cells divided by DAPI cells. To assess Aβ plaque and HSV-GFP phagocytosis by microglia cells, confocal single-plane images of lysosomes (CD68), Aβ and microglia (Iba1) were separately isolated, and the areas of colocalization (CD68^+^Aβ^+^GFP^+^ or Iba1^+^Aβ^+^GFP^+^) were measured by ImageJ software. Image processing and analysis were performed by Image J software or Imaris software (Bitplane, Switzerland), as appropriate. Imaris were used for 3D reconstruction of confocal images to analyze microglia morphology and colocalization of Aβ plaque with CD68, GFP with CD68, Aβ plaque with Iba1, and GFP with Iba1.

### Immunoblotting

Mouse brain tissues were homogenized and lysed in RIPA lysis buffer (P0013B, Beyotime) supplemented with PMSF (ST505, Beyotime) on ice for 30 min. Subsequently, the lysates were separated on SDS-PAGE gels and transferred to PVDF membranes. The membranes were blocked in 5% skim milk in TBS-T for 2 h and incubated with specific primary antibodies against Caspase1 (p20) (1:1000, Cat #AG-20B-0042, Adipogen, PRID: AB_2490248), beta Amyloid (APP, 1:1000, Cat #51-2700, Thermo Fisher, PRID: AB_2533902), presenilin1/PS-1 (1:1000, Cat #ab76083, Abcam, PRID: AB_1310605), ADAM10 (1:1000, Cat #ab124695, Abcam, PRID: AB_10972023), BACE1 (1:1000, Cat #ab2077, Abcam, PRID: AB_302817), p-TBK1 Ser^172^ (D52C2) (1:1500, Cat #5483, Cell Signaling Technology, PRID: AB_10693472), TBK1/NAK (1:1500, Cat #3504, Cell Signaling Technology, PRID: AB_2255663), p-STING Ser^366^ (1:1500, Cat #85735, Cell Signaling Technology, PRID: AB_2732796), STING (D2P2F) (1:1000, Cat #13647, Cell Signaling Technology, PRID: AB_2732796), GAPDH (14C10) (1:3000, Cat #2118, Cell Signaling Technology, PRID: AB_561053) overnight at 4 °C. After three washes with TBS-T, the membranes were incubated with HRP-conjugated secondary antibodies for 2 h at room temperature. Immunoreactive bands were imaged using the Automatic Chemiluminescence Imaging System (Tanon 5200).

### RNA isolation and quantitative real-time PCR (qRT-PCR)

PBS or HSV-GFP (1 × 10^5^ PFUs/hippocampus) was administered into 3-month-old 5xFAD mice. Three and seven days after HSV-1 infection, control and HSV-1-infected hippocampi from the same mice were collected and homogenized in TRIZOL reagent to isolate total RNA. Subsequently, cDNA was reverse transcribed using the One-Step gDNA Removal and cDNA Synthesis Kit (Mei5Bio). Relative mRNA expression of *IL6*, *IL-1β*, *TNFα*, *IFNβ*, *IFNγ*, *IL10*, and *CCL5* was determined by quantitative real-time PCR.

Real-time PCR was performed using SYBR Green Master Mix with the following primers: IL6 (Fp: 5′-TACCACTTCACAAGTCGGAGGC-3′; RP: 5′-CTGCAAGTGCATCATCGTTGTTC-3′), IL-1β (Fp: 5′-TGGACCTTCCAGGATGAGGACA-3′; Rp: 5′-GTTCATCTCGGAGCCTGTAGTG-3′), TNFα (Fp: 5′-GGTGCCTATGTCTCAGCCTCTT-3′; Rp: 5′-GCCATAGAACTGATGAGAGGGAG-3′), IFNβ (5′-GCCTTTGCCATCCAAGAGATGC-3′ Rp: 5′-ACACTGTCTGCTGGTGGAGTTC-3′), IFNγ (Fp: 5′-CAGCAACAGCAAGGCGAAAAAGG-3′; Rp: 5′-TTTCCGCTTCCTGAGGCTGGAT-3′), IL10 (Fp: 5′-CGGGAAGACAATAACTGCACCC-3′; Rp: 5′-CGGTTAGCAGTATGTTGTCCAGC-3′), CCL5 (Fp: 5′-CCTGCTGCTTTGCCTACCTCTC-3′; Rp: 5′-ACACACTTGGCGGTTCCTTCGA-3′) and β-actin (Fp: 5′-CATTGCTGACAGGATGCAGAAGG-3′; Rp: 5′- TGCTGGAAGGTGGACAGTGAGG-3′). The expression levels of genes were normalized to β-actin and quantified by the ΔΔCT method.

### Behavioral tests

Mice were housed in groups of 3–5 animals on a 12:12 h light–dark cycle. 3-month-old male 5xFAD mice, which were infected with sterile PBS or HSV-1-GFP, were used for all the behavioral tests. Videos were recorded and analyzed by the Smart V3.0.03 software (Panlab, Barcelona, Spain).

#### Y-maze test

Y-maze spontaneous alteration test was carried out to assess short-term spatial memory. Mice were allowed to acclimate to the testing room for 48 h. The Y-maze apparatus comprised three opaque arms at 120° angles from each other. Each mouse was gently placed in the distal part of the maze and allowed to explore freely for 5 min. Detailed recordings were made of their movements and the total number of arms entered. Spontaneous Alteration [%] was defined as the ratio of consecutive entries into 3 different arms divided by the total arm entries minus 2.

#### Morris water maze test

A circular water tank (diameter: 120 cm) was filled with water to a depth of 25 cm, and the water was made opaque with non-toxic white paint. A 13-cm in diameter round platform was hidden 1 cm beneath the surface of the water at the center of a given quadrant of the water tank. Mice received training for five consecutive days, with each session comprising four trails starting from different sites. For each trail, the mouse was gently released from the wall of the tank and allowed to explore, locate and stand on the platform for a duration of 20 s, within the 60-s trail period. Following the completion of training, a probe test was performed 24 h later. During the probe test, the platform was removed and task performance including swimming tracks, speed, time spent and entries into the platform were recorded for analysis.

### Flow cytometry

For the Aβ and HSV-GFP phagocytosis assay, HT22 cells were infected with HSV-1-GFP for 24 h, then washed and suspended with PBS. BV2 and primary microglial cells were incubated with 1 μg/ml Alexa 555-labeled Aβ_1-42_, or 1 μg/ml Alexa 555-labeled Aβ_1-42_ plus HSV-1-GFP from HT-22 neuronal cells for 4 h. Subsequently, cells were harvested and analyzed using a FACSAria (BD Bioscience). These experiments were repeated three times and analyzed using FlowJo v10.4 (Tree Star).

### Drug treatment

PLX3397 (Selleck, Cat# S7818) was employed for the pharmacological depletion of brain microglia cells. Briefly, mice were fed with PLX3397-formulated AIN-76A diet (290 mg/kg) or normal AIN-76A diet ad libitum [41], and the treatment continued until the mice were euthanized.

MCC950 sodium (MedChemExpress, MCE, Cat# HY-12815A) is a selective NLRP3 inhibitor. Mice received MCC950 (10 mg/kg, dissolved in PBS with 2% DMSO) or vehicle (PBS with 2% DMSO) via intraperitoneal injection every second day after HSV-1 infection for 21 days.

### ELISA assay

PBS or HSV-GFP (1 × 10^5^ PFUs/hippocampus) was injected into 1-month-old, 3-month-old, and 6-month-old 5xFAD mice. Seven days later, hippocampi were collected, homogenized, and diluted in PBS. The concentration of IL-1β was assessed using ELISA kits according to the manufacturer’s instructions (EK0394, BOSTER Biological Technology).

### Statistical analysis

All statistical analyses were performed using Prism 8.0 (GraphPad Software). Datasets were analyzed for p-values using either unpaired Student’s two-tailed *t* tests or ANOVA multiple comparison post hoc tests; all data are presented as means ± SEM. Statistical significance was represented as follows: *p ≤ 0.05, **p ≤ 0.01, ***p ≤ 0.001, ****p ≤ 0.0001, n.s.: *p* > 0.05.

## Results

### HSV-1 infection exacerbates Aβ plaque deposition and cognitive decline

**Fig. 1:**
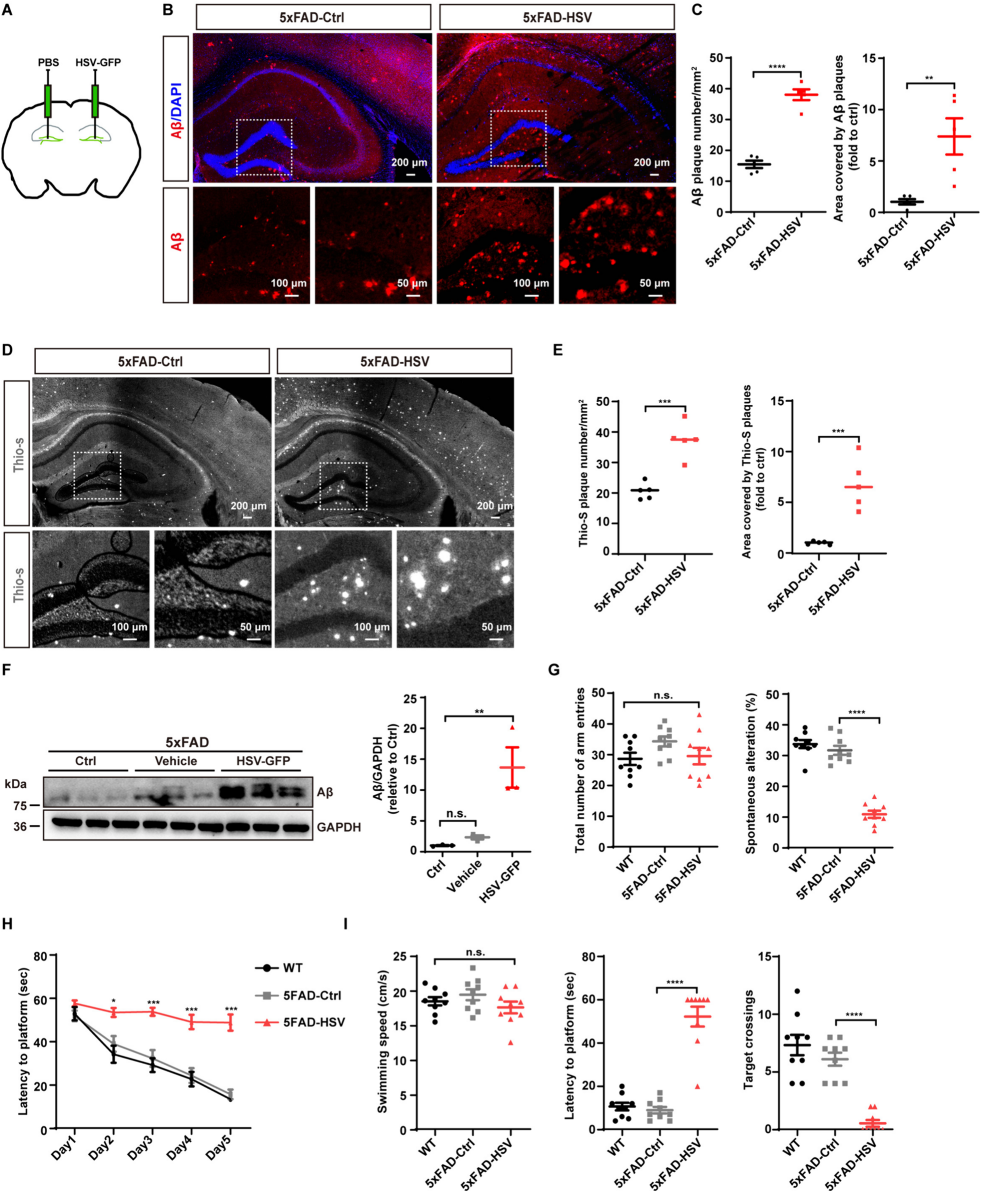
HSV-1 infection exacerbates Aβ deposition and cognitive decline in 5xFAD mice. **A** Three-month-old 5xFAD mice were injected with 1 × 10^5^ PFUs of HSV-GFP or PBS in the hippocampus of the brain. **B** Representative images of Aβ (MAOB2, red) in the whole brain of 3-month-old 5xFAD mice seven days after HSV-GFP infection. Scale bars, 200 μm, 100 μm and 50 μm. **C** Quantification of Aβ plaques and plaque area in the brain of 3-month-old 5xFAD mice infected with PBS or HSV-GFP. (*n* = 5 mice per group). **D** Representative images of Thioflavin S staining (gray) in the whole brain of 3-month-old 5xFAD mice seven days after HSV-GFP infection. Scale bars, 200 μm, 100 μm and 50 μm. **E** Quantification of Aβ plaques and plaque area in the brain of 3-month-old 5xFAD mice infected with PBS or HSV-GFP. (*n* = 5 mice per group). **F** Three-month-old 5xFAD mice were randomly divided to three groups: control group (no treatment); Vehicle group (PBS treatment) and HSV-GFP group. Mouse hippocampi were collected and homogenized for immunoblotting of Aβ seven days post-infection. The intensities of Aβ were quantified by ImageJ software. **G** Three-month-old 5xFAD mice were randomly divided to two groups: control group (PBS treatment) and HSV-GFP group. Y-maze test was performed to measure the total number of arm entries and spontaneous alteration (%) at 21 days post-infection. (*n* = 9 mice per group). **H**, **I** Morris water maze was performed to evaluate the spatial learning and memory of 5xFAD mice infected with HSV-GFP. During the training phase, each group showed improved latency to the platform, but compared with the control group, the HSV-GFP group exhibited a significant delay to locate the platform (**H**). In the probe test, there was no significant difference in swimming speeds between the two groups of the mice. Compared to the control group, the 5xFAD-HSV group exhibited a significantly longer latency to locate the platform, but fewer target crossings (**I**). (*n* = 9 mice per group). Data are presented as means ± SEM. n.s.: *p* > 0.05, *p ≤ 0.05, **p ≤ 0.01, ***p ≤ 0.001, and ****p ≤ 0.0001

To investigate the correlation between HSV infection and Aβ plaque deposition, we administrated HSV-1-GFP virus into the hippocampus of 3-month-old transgenic AD (5xFAD) mice, using phosphate-buffered saline (PBS) as a vehicle control (Fig. 1A). Seven days after stereotaxic injection of HSV-1-GFP, results of Aβ immunostaining revealed a dramatic increase of amyloid plaques in the whole brain (Fig. 1B and C). Thio-S staining of brain slices confirmed a significant increase in both the number and size of Aβ plaques in 3-month-old 5xFAD mice infected with HSV-GFP compared to those injected with PBS (Fig. 1D and E). The Aβ protein amounts in the hippocampus of 3-month-old 5xFAD-HSV mice were notably higher compared to 3-month-old 5xFAD-Ctrl mice (Fig. 1F). Accumulation and deposition of Aβ may trigger neurotoxic effects, neuronal loss, and degeneration, ultimately leading to cognitive impairments. To assess the detrimental effects of HSV-1 infection on amyloid deposition and its association with cognitive dysfunction in 5xFAD mice, we performed Y-maze and Morris water-maze tests in 3-month-old 5xFAD mice following virus injection. At 3 months of age, 5xFAD mice did not exhibit significant deficits in spatial learning and memory (Fig. 1G–I). However, HSV-1 infection resulted in significant impairments in spatial learning and memory, characterized by reduced spontaneous alterations without a decrease in total arm entries (Fig. 1G), prolonged escape latency time during the training trails and the probe trail, and a lower number of platform crossings (Fig. 1H and I). Swimming speeds were comparable across all groups of animals, thus excluding impairments in motor function (Fig. 1I).

**Fig. 2:**
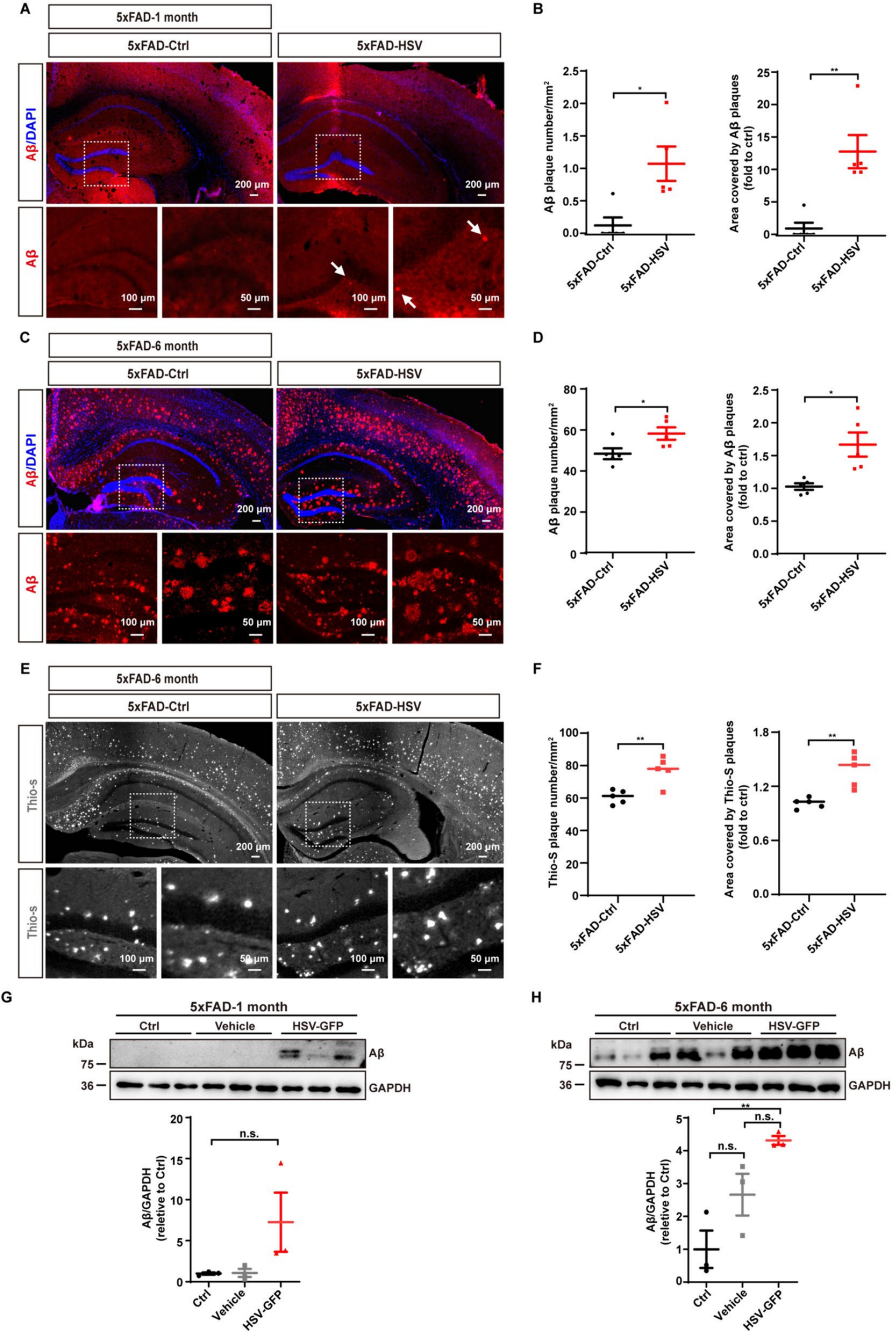
HSV-1 infection exacerbates Aβ deposition in 1-month-old and 6-month-old 5xFAD mice. **A** and **B** Representative images (**A**) and quantification of Aβ plaques and plaque area (**B**) in the whole brain and hippocampus of 1-month-old 5xFAD mice seven days after HSV-GFP infection. Scale bars, 200 μm, 100 μm and 50 μm. (*n* = 5 mice per group). **C** and **D** Representative images (**C**) and quantification of Aβ plaques and plaque area (**D**) in the whole brain of 6-month-old 5xFAD mice seven days after HSV-GFP infection. Scale bars, 200 μm, 100 μm and 50 μm. (*n* = 5 mice per group). **E** Representative images (**E**) and quantification of Aβ plaques and plaque area (**F**) in the whole brain of 6-month-old 5xFAD mice seven days after HSV-GFP infection. Scale bars, 200 μm, 100 μm and 50 μm. (*n* = 5 mice per group). **G** Immunoblotting and quantification of Aβ protein levels in the hippocampus of 1-month-old 5xFAD mice (Ctrl), 5xFAD mice infected with PBS (Vehicle) or 5xFAD mice seven days after HSV-GFP infection. (*n* = 3 mice per group). **H** Immunoblotting and quantification of Aβ protein levels in the hippocampus of 6-month-old 5xFAD mice (Ctrl), 5xFAD mice infected with PBS (Vehicle) or 5xFAD mice seven days after HSV-GFP infection. (*n* = 3 mice per group). All data are presented as means ± SEM. n.s.: *p* > 0.05, *p ≤ 0.05, **p ≤ 0.01

To further confirm that HSV-1 facilitate Aβ accumulation, we also administrated HSV-1-GFP virus into the hippocampus of 1-month-old and 6-month-old 5xFAD mice and observed a significant increase in the number and size of Aβ plaques in 1-month-old 5xFAD-HSV mice (Fig. 2A and B) and 6-month-old 5xFAD-HSV mice (Fig. 2C and D). Of note, Aβ deposition was already clearly detectable in 6-month-old 5xFAD-Ctrl mice, whereas the density and signal intensity of Aβ plaques were stronger in 6-month-old 5xFAD-HSV mice (Fig. 2E and F). The Aβ protein amounts in the hippocampus also increased in both 1-month-old 5xFAD-HSV mice (Fig. 2G) and 6-month-old 5xFAD-HSV mice (Fig. 2H). Together, these data indicate that HSV-1 infection can exacerbate β-amyloid deposition and contribute to spatial and learning memory deficits in 5xFAD mice.

### HSV-1 infection promotes microglia cell activation in 5xFAD mice

**Fig. 3:**
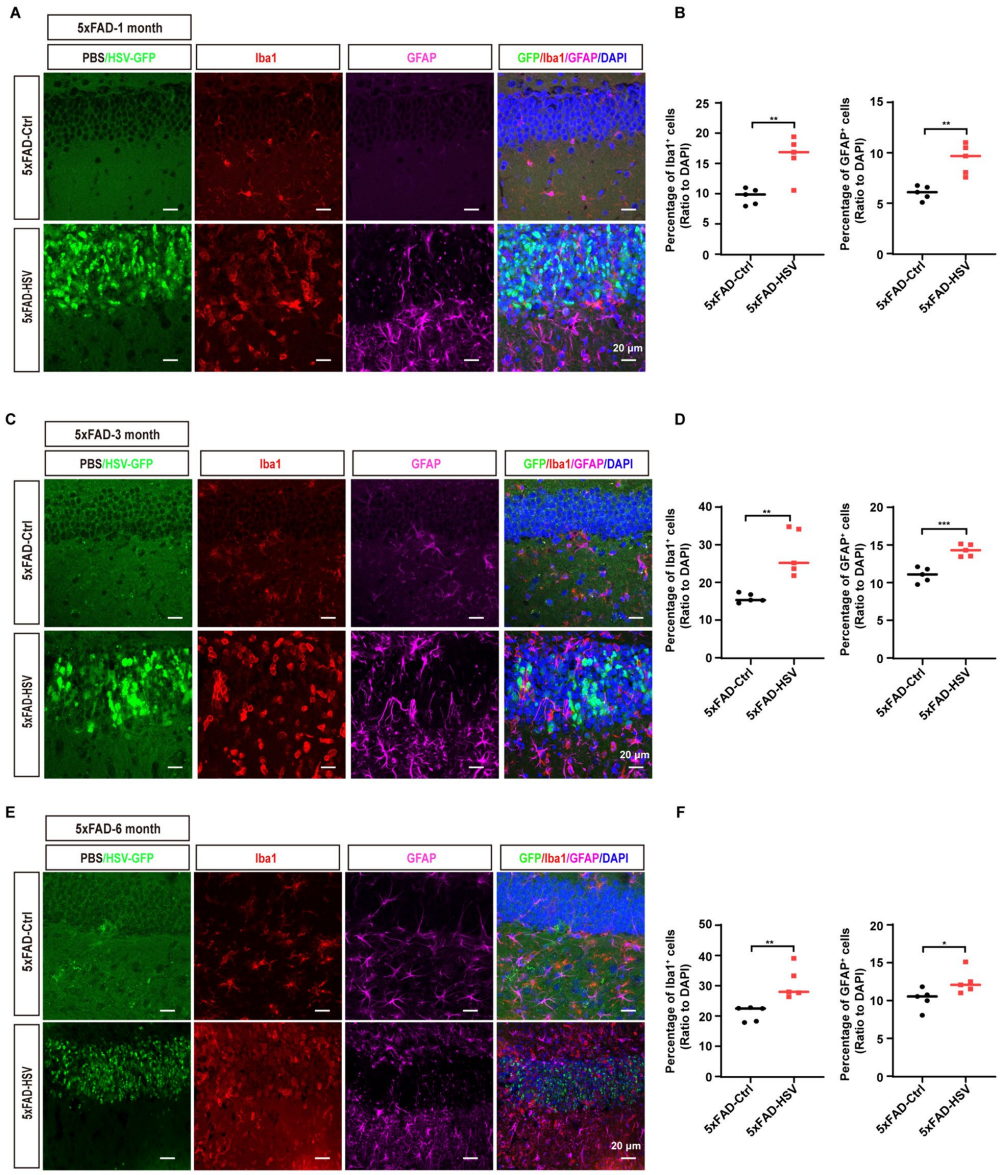
HSV-1 infection leads to gliosis in 5xFAD mice. **A** Representative images of microglia (Iba1, red) and astrocytes (GFAP, magenta) in the hippocampus of 1-month-old 5xFAD mice seven days after HSV-GFP infection. Scale bars, 20 μm. **B** Quantification of Iba1^+^ microglia and GFAP^+^ astrocytes in the hippocampus of 1-month-old 5xFAD mice seven days after HSV-GFP infection. (*n* = 5 mice per group). **C** Representative images of microglia (Iba1, red) and astrocytes (GFAP, magenta) in the hippocampus of 3-month-old 5xFAD mice seven days after HSV-GFP infection. Scale bars, 20 μm. **D** Quantification of Iba1^+^ microglia and GFAP^+^ astrocytes in the hippocampus of 3-month-old 5xFAD mice seven days after HSV-GFP infection. (*n* = 5 mice per group). **E** Representative images of microglia (Iba1, red) and astrocytes (GFAP, magenta) in the hippocampus of 6-month-old 5xFAD mice seven days after HSV-GFP infection. Scale bars, 20 μm. **F** Quantification of Iba1^+^ microglia and GFAP^+^ astrocytes in the hippocampus of 6-month-old 5xFAD mice seven days after HSV-GFP infection. (*n* = 5 mice per group). All data are presented as means ± SEM. *p ≤ 0.05, **p ≤ 0.01, and ***p ≤ 0.001

**Fig. 4:**
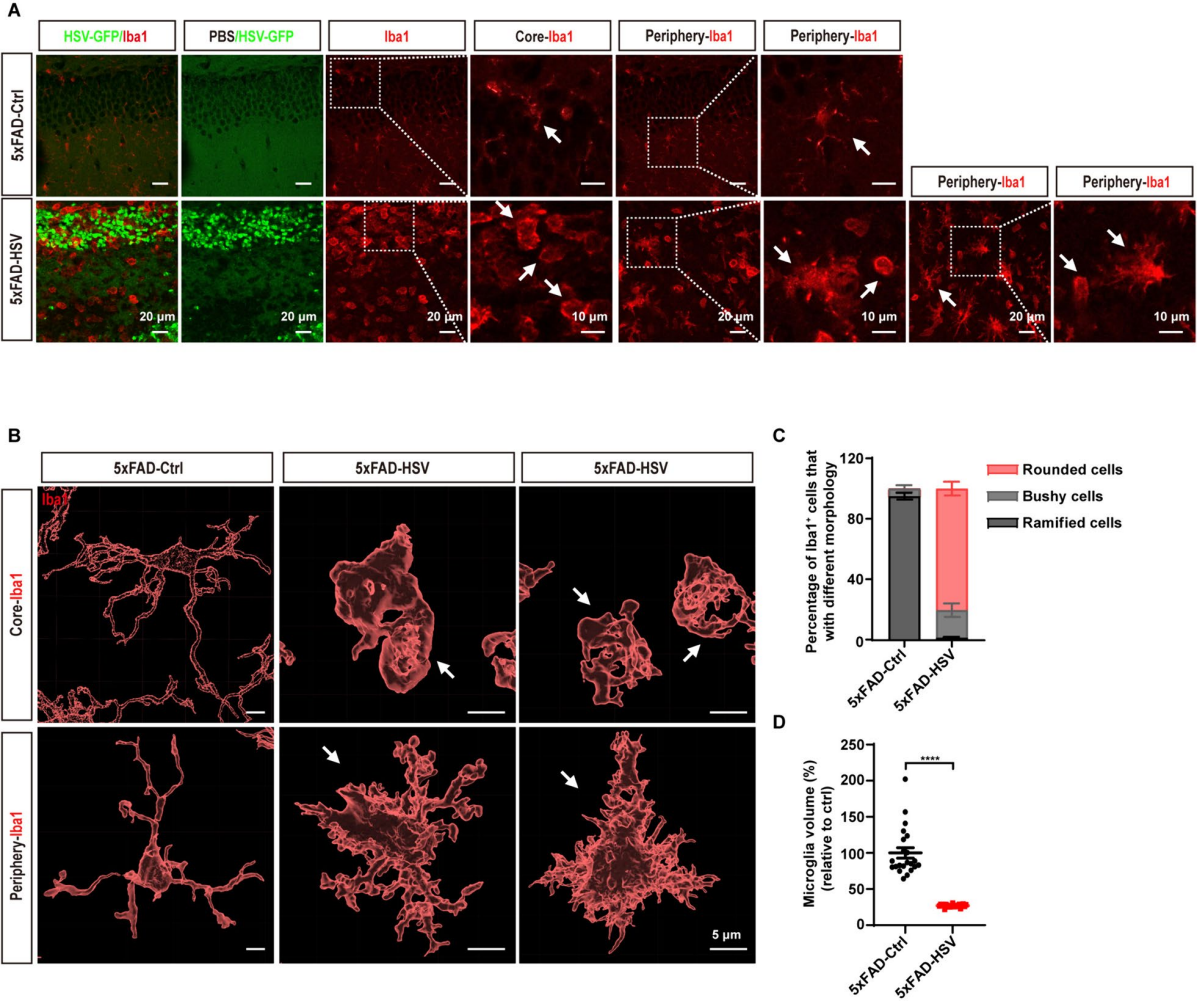
HSV-1 infection promotes microglia activation in the viral core in 5xFAD mice. **A** Representative images of Iba1^+^ microglia in the core and periphery of hippocampus seven days after HSV-GFP infection. Dashed-white frames are magnified to illustrate the representative morphology of microglia. Original magnification × 40, scale bars; 20 μm. Zoom-in images with a scale bar equal to 10 μm. **B** Three-dimensional reconstruction of Iba1^+^ microglia in the core and periphery of the hippocampus seven days after HSV-GFP infection. Scale bars, 5 μm. **C** and **D** Quantification of microglial morphology (**C**) and processes (**D**) in the 5xFAD mice seven days after HSV-GFP infection. (*n* = 5 mice per group). All data are presented as means ± SEM. ****p ≤ 0.0001

The proliferation and activation of glial cells are prominent features of virus infection [42, 43]. Iba1 and GFAP are widely used as immunohistochemical markers for microglia and astrocytes, and their expression increases upon glial cell activation. Indeed, we observed increasing expression of Iba1 and GFAP in the hippocampus of 5xFAD mice infected with HSV-1, regardless of their age (Fig. 3). Interestingly, we observed distinct microglia morphology that may represent different activation states following HSV-1 infection. In the viral core, microglia often displayed an activated “rounded” morphology, while in the periphery of the viral core, microglia displayed an activated “ameboid” morphology characterized by shorter processes, larger cell bodies and reduced microglia volumes (Fig. 4A). In contrast, microglia exhibited a ramified morphology in 5xFAD-Ctrl mice (Fig. 4B and C). Collectively, our data suggest that HSV-1 infection enhances gliosis and microglial activation in 5xFAD mice.

### HSV-1 infection induces enhanced microglial phagocytosis of HSV-GFP-positive neuronal cells

The production and aggregation of Aβ are attributed to the imbalance between Aβ production and clearance [44]. Having observed significant Aβ plaque deposition in 5xFAD mice after HSV-1 infection, we next investigated whether APP processing was involved in Aβ production. To test this hypothesis, we examined a series of key factors involved in Aβ production. In comparison to 5xFAD control mice, there was no significant change in the expression levels of APP, PS1, or the APP processing secretases ADAM10 and BACE1 in the HSV-infected 5xFAD mice (Figure S1). These results demonstrate that HSV-1 infection exacerbates Aβ burden without altering APP expression and processing, both in young and aged 5xFAD mice.

**Fig. 5:**
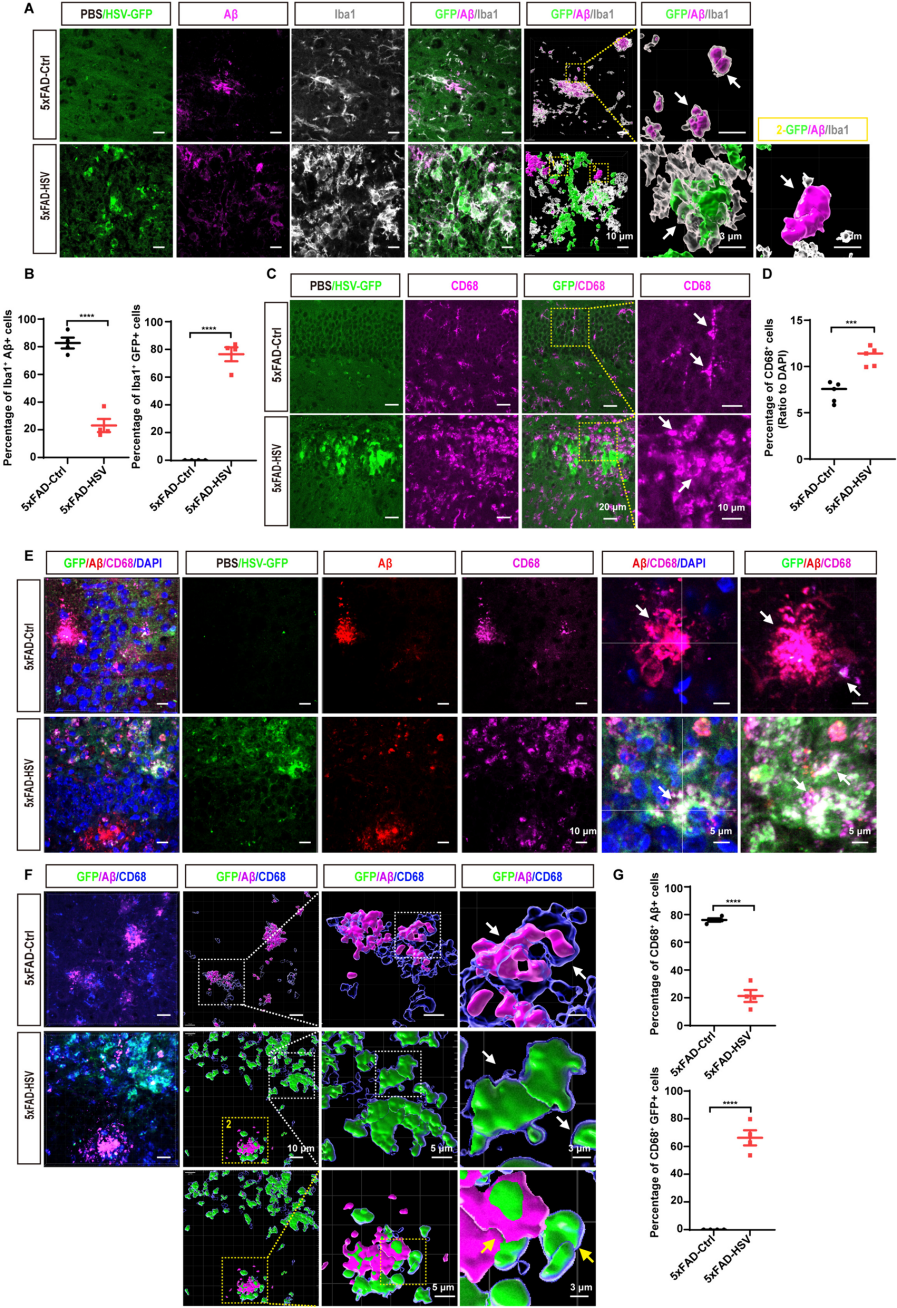
HSV-1 infection induces enhanced microglial phagocytosis of HSV-GFP-positive neuronal cells. **A** Representative images of microglia (Iba1, gray) and Aβ plaques (magenta) in the hippocampus of 3-month-old 5xFAD mice seven days after HSV-GFP infection. Right panels show three-dimensional reconstructed enlarged images of yellow dotted boxes, illustrating engulfed GFP-positive cells or Aβ plaques by microglia. Scale bars, 10 μm and 3 μm. **B** Quantification of engulfed Aβ plaques or HSV-GFP-positive cells by microglia, indicated by the co-staining of Aβ and Iba1 (Aβ^+^Iba1^+^) or GFP and Iba1 (GFP^+^Iba1^+^). (*n* = 4 mice per group). **C** and **D** Representative images (**C**) and quantification (**D**) of CD68-positive cells in the hippocampus of 3-month-old 5xFAD mice seven days after HSV-GFP infection. Original magnification × 40, scale bars; 20 μm. Zoom-in images with a scale bar equal to 10 μm. (*n* = 5 mice per group). **E** Representative images of phagocytic microglia (CD68, magenta) co-stained with Aβ (red) in the hippocampus of 3-month-old 5xFAD mice (Ctrl) or 5xFAD mice infected with HSV-GFP (HSV). Original magnification × 63, scale bars; 10 μm. Zoom-in images with a scale bar equal to 5 μm. **F** Representative images of phagocytic microglia (CD68, blue) co-stained with Aβ plaques (magenta) in the core of the hippocampus infected with PBS or HSV-GFP. Three-dimensional reconstructed enlarged images of dashed-white or dashed-yellow frames show engulfed GFP-positive cells or Aβ plaques in the lysosomes (blue). Original magnification × 63, scale bars; 10 μm. Zoom-in images with a scale bar equal to 5 μm and 3 μm. **G** Quantification of engulfed GFP-positive cells or Aβ plaques in **F**. (*n* = 4 mice per group). All data are presented as means ± SEM. ***p ≤ 0.001, ****p ≤ 0.0001

Given the significant accumulation of Aβ plaques with unchanged Aβ production in 5xFAD mice after HSV-1 infection, we next sought to explore whether HSV-1 infection affects Aβ clearance. Since microglia play a crucial role in phagocytizing and degrading extracellular Aβ in the brain of AD mice, we then performed immunostaining assay using anti-Aβ antibody along with anti-Iba1 antibody to visualize the colocalization of Aβ and microglia. Confocal images showed that microglia clustered around the Aβ plaques in 5xFAD-Ctrl mice (Fig. 5A). However, we observed a significant decrease in the number of Iba1 + microglia near Aβ plaques and a significant increase in the number of Iba1 + microglia near the viral core in 5xFAD mice following HSV-1 infection (Fig. 5A and B).

**Fig. 6:**
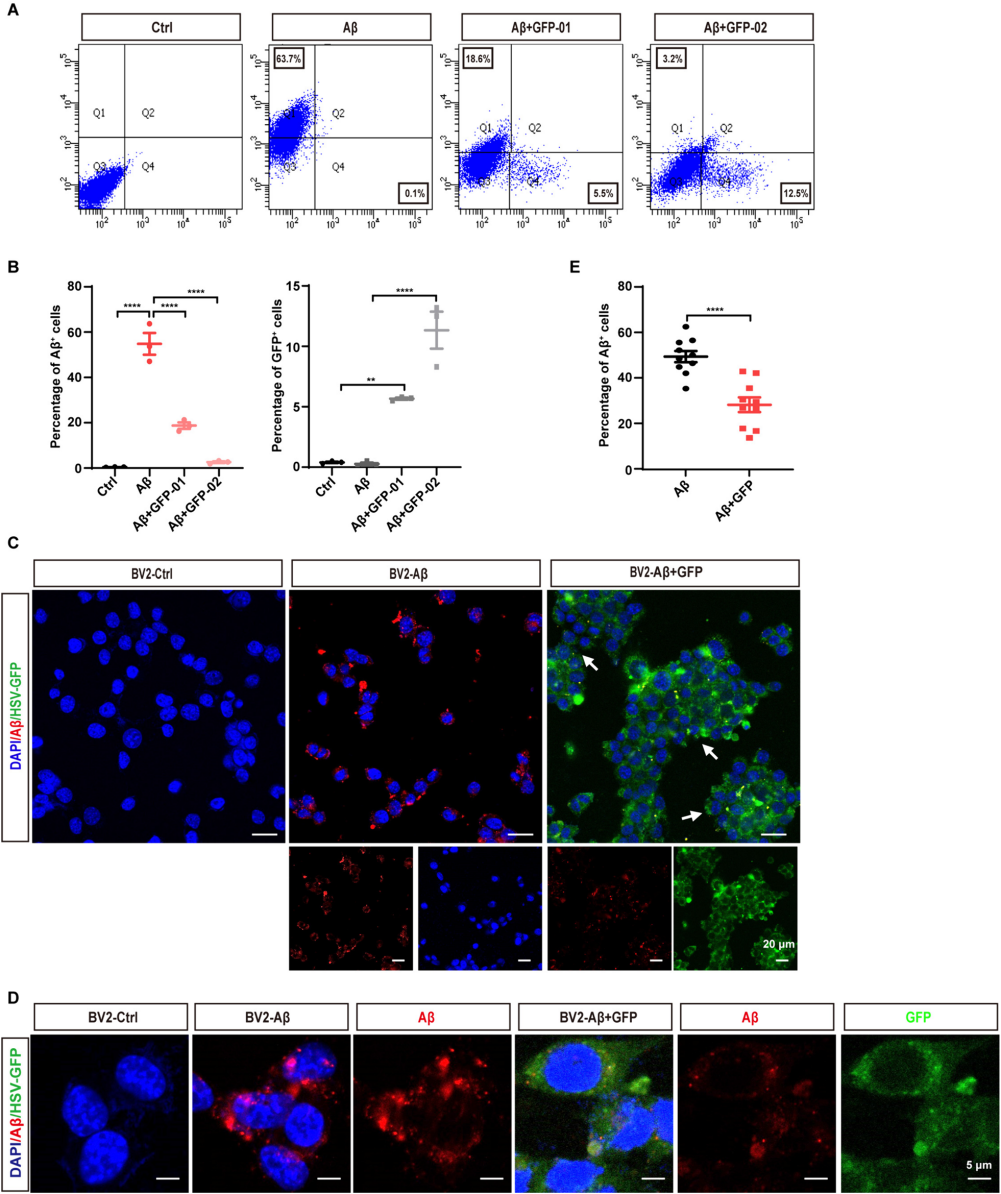
HSV-1 infection induces enhanced microglial phagocytosis of HSV-GFP-positive neuronal cells in vitro. **A** Representative FACS dot plots of engulfed GFP-positive cell fragments or 555-labed Aβ in BV2 microglial cells are shown. These experiments are repeated three times. **B** Quantification of engulfed GFP-positive cell fragments or 555-labed Aβ in the BV2 microglial cells as measured by flow cytometry. **C** Representative images of microglial phagocytosis of GFP-positive cell fragments or 555-labed Aβ after uptake for 4 h in cultured BV2 microglial cells. These experiments are repeated three times. Scale bars; 20 μm. **D** Representative images of microglial phagocytosis of GFP-positive cell fragments or 555-labed Aβ after uptake for 4 h in cultured BV2 microglial cells. These experiments are repeated three times. Scale bars; 5 μm. **E** Quantification of internalized 555-labed Aβ using ImageJ software. (*n* = 10 per group). All data are presented as means ± SEM. **p ≤ 0.01, ****p ≤ 0.0001

To further elucidate whether the enhanced recruitment of microglia around the viral core affects Aβ engulfment and clearance, we performed immunostaining of CD68, a phagocytic marker for microglia. Compared with the control group, HSV-1 infection markedly upregulated CD68 expression in all age groups of 5xFAD mice (Fig. 5C and D, Figure S2A and S2B). Quantitative analysis of the colocalization area of CD68 with Aβ or HSV-GFP revealed a significant increase in GFP^+^CD68^+^ microglia and a significant decrease in Aβ^+^CD68^+^ microglia in 5xFAD-HSV mice (Fig. 5E–G, Figure S2C), This suggests that microglia preferentially phagocytize HSV-GFP-positive neuronal cells after HSV-1 infection, but not Aβ plaques, leading to Aβ accumulation in 5xFAD-HSV mice. To further characterize this microglia-HSV-1 module, we examined the effects of HSV-1 infection on Aβ uptake in cultured BV2 and primary microglial cells by flow cytometry. BV2 and primary microglial cells were incubated with Alexa 555-labeled Aβ_1-42_ or Alexa 555-labeled Aβ_1-42_ plus HSV-1-GFP from HT-22 neuronal cells for 4 h. Consistent with our in vivo data, we observed a decrease in Aβ_1-42_ uptake and an increase in GFP-positive phagocytosis upon incubation with GFP-positive cell debris (Fig. 6A, B, Figure S3A-B). In addition, we confirmed this phenomenon using an immunostaining assay, which consistently showed that BV2 and primary microglial cells preferred to phagocytize GFP-positive cell debris (Fig. 6C–E, Figure S3C-D). Together, these results suggest that HSV-1 infection enhances the recruitment of microglia to the viral core and decreases their phagocytic uptake of Aβ plaques, consequently leading to Aβ accumulation and deposition both in vivo and in vitro.

### Microglia are indispensable to control Aβ plaque aggregation after HSV-1 infection

**Fig. 7:**
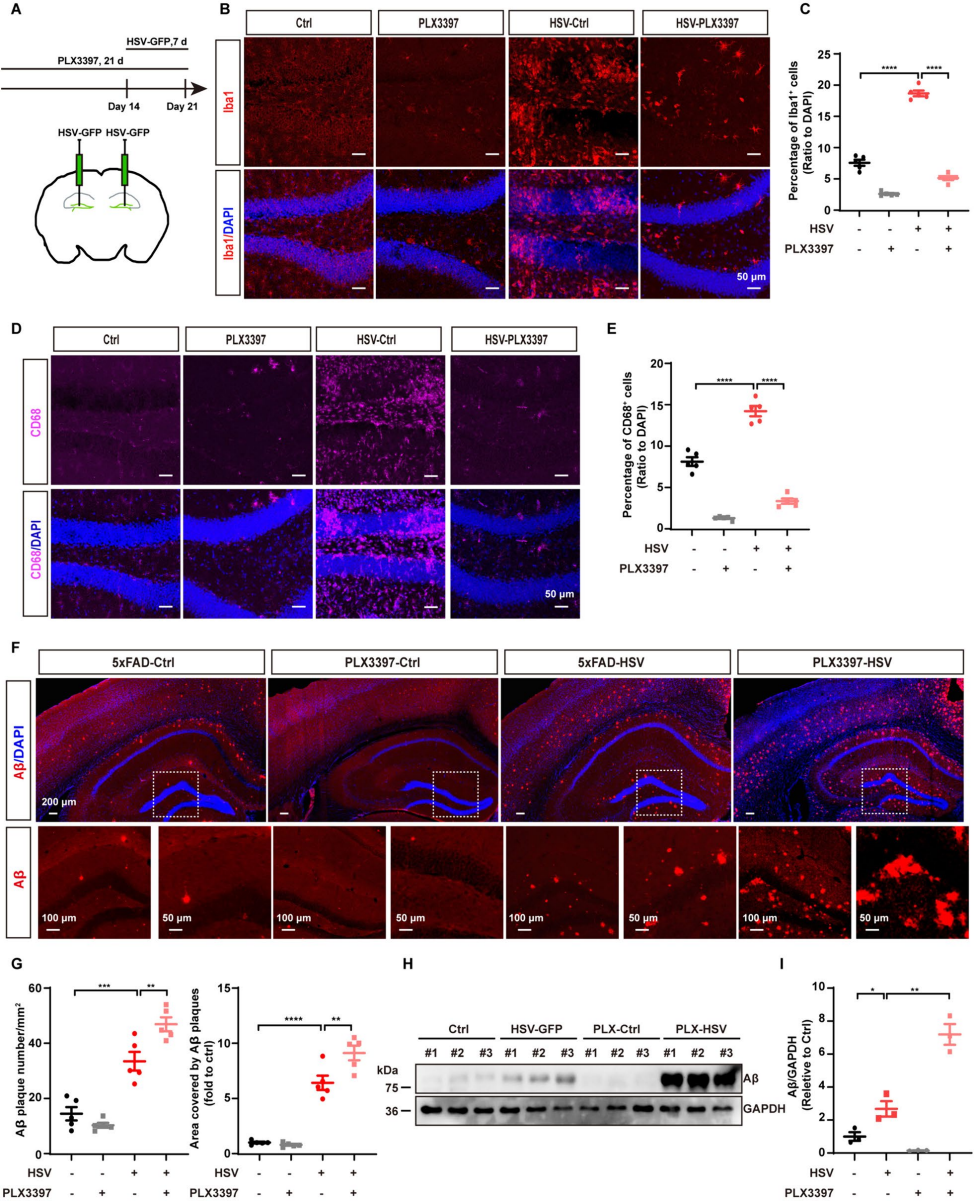
Depletion of microglia increases Aβ plaque deposition and reduces microglial phagocytosis after HSV-1 infection. **A** Scheme for PLX3397 administration and HSV-GFP injection in 5xFAD mice. **B** and **C** Representative images (**B**) and quantification of Iba1-positive microglial cells (**C**) in the hippocampus of 3-month-old 5xFAD mice seven days after HSV-GFP infection following 21 days treatment with PLX3397. Scale bars, 50 μm. (*n* = 5 mice per group). **D** and **E** Representative images (**D**) and quantification of CD68-positive microglial cells (**E**) in the hippocampus of 3-month-old 5xFAD mice seven days after HSV-GFP infection following 21 days treatment with PLX3397. Scale bars, 50 μm. (*n* = 5 mice per group). **F** and **G** Representative images (**F**) and quantification of Aβ plaques and plaque area (**G**) in the whole brain of 3-month-old 5xFAD mice seven days after HSV-GFP infection following 21 days treatment with PLX3397. Scale bars, 200 μm, 100 μm and 50 μm. (*n* = 5 mice per group). **H** and **I** Immunoblotting and quantification of Aβ protein in the hippocampus of 3-month-old 5xFAD mice seven days after HSV-GFP infection following 21 days treatment with PLX3397. Data are presented as means ± SEM. *p ≤ 0.05, **p ≤ 0.01, ***p ≤ 0.001, and ****p ≤ 0.0001

Our results indicated that microglia may be not able to perform normal Aβ clearance following HSV-1 infection. To further confirm the role of microglia in Aβ pathology, we employed the CSF1R inhibitor, PLX3397, to deplete microglia [45, 46]. 5xFAD mice were fed with PLX3397-formulated diet for 14 days and then injected with PBS or HSV-GFP for another 7 days (Fig. 7A). After PLX3397 treatment, both microglia numbers and their phagocytic ability, as assessed by Iba1 and CD68 staining, were substantially reduced in both 5xFAD-Ctrl mice and 5xFAD-HSV mice (Fig. 7B–E, Figure S4). To determine if microglia are responsible for HSV-1-mediated plaque aggregation, we depleted microglia with PLX3397, followed by the administration of HSV-1 to the hippocampus. Consistent with the previous findings, treatment of 5xFAD mice with PLX3397 led to a reduction in Aβ plaque deposition (Fig. 7F, G and Figure S5). However, compared to 5xFAD-HSV mice in the absence of PLX3397 treatment, the plaque load was markedly increased in 5xFAD-HSV mice treated with PLX3397 (Fig. 7F, G, Figure S5), indicating that microglia play an indispensable role in controlling HSV-1-mediated Aβ plaque aggregation. Furthermore, we examined the protein amounts of Aβ in 5xFAD mice treated with PLX3397 and injected with HSV-1 and found that the amounts of Aβ were significantly increased in 5xFAD-HSV mice treated with PLX3397 (Fig. 7H, I). Together, these results indicate that microglia play an indispensable role in maintain the homeostasis of HSV-1-GFP and Aβ plaque aggregation in 5xFAD-HSV mice.

### NLRP3 inflammasome is activated after HSV-1 infection in 5xFAD mice

**Fig. 8:**
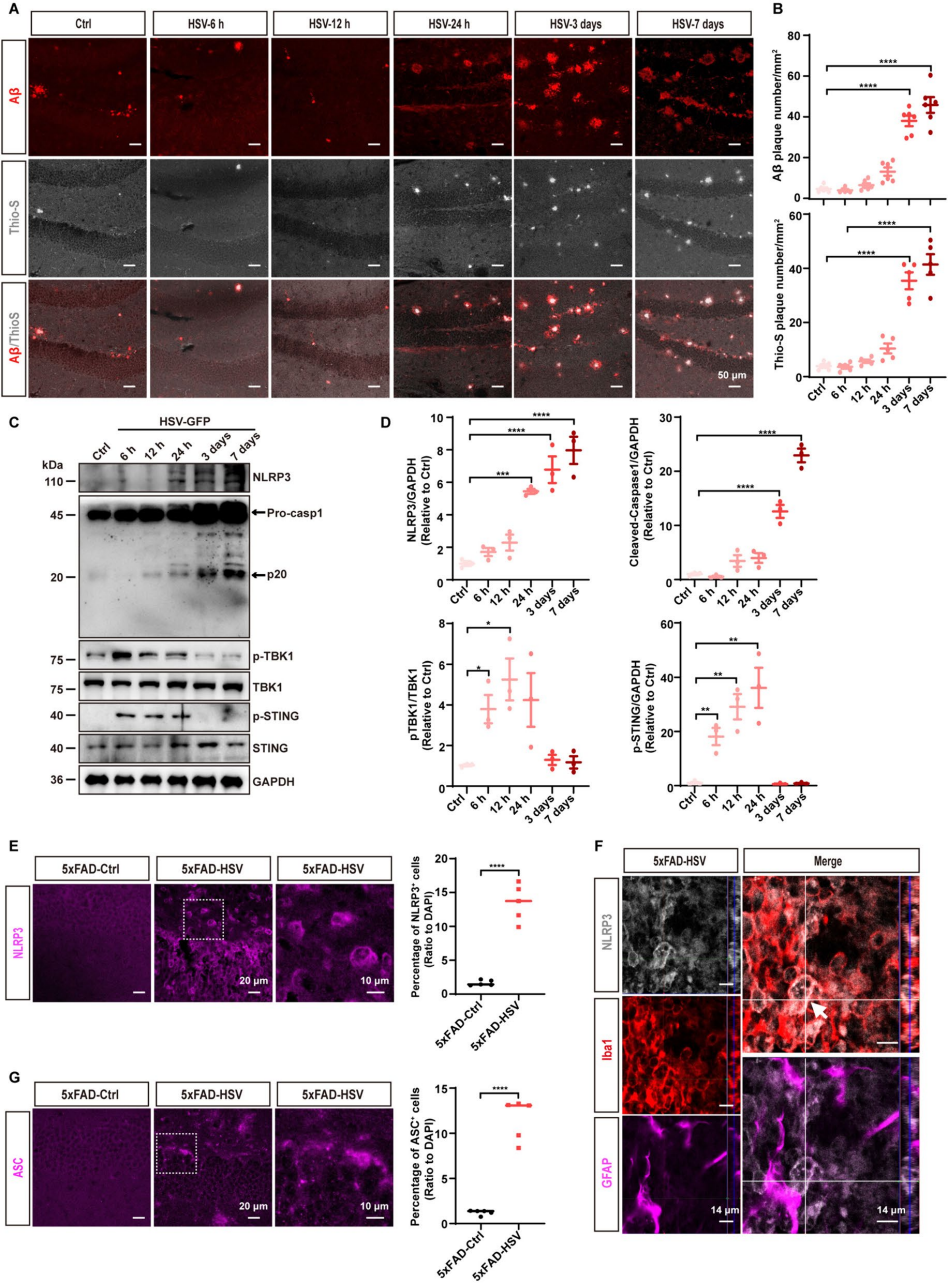
NLRP3 inflammasome is activated after HSV-1 infection in 5xFAD mice. **A** Representative images of Aβ (red) and Thio-S (gray) in the hippocampus of 3-month-old 5xFAD mice infected with PBS or HSV-GFP over the indicated time periods. **B** Quantification of Aβ plaques in the hippocampus of 3-month-old 5xFAD mice infected with PBS or HSV-GFP over the indicated time periods. (*n* = 5 mice per group). **C** Western blot analysis of NLPR3 inflammasome protein markers and cGAS-STING signaling pathway protein markers from the hippocampi over the indicated time periods upon HSV-GFP infection. **D** Quantification of the band intensity in **C** using ImageJ software. (*n* = 3 mice per group). **E** Representative images and quantification of NLRP3-positive cells (magenta) in the hippocampus of 3-month-old 5xFAD mice seven days after HSV-GFP infection. Original magnification × 40, scale bars; 20 μm. Zoom-in images with a scale bar equal to 10 μm. (*n* = 5 mice per group). **F** Representative images of NLRP3 (gray) co-stained with microglia (Iba1, red) and astrocytes (GFAP, magenta) in the hippocampus of 3-month-old 5xFAD mice seven days after HSV-GFP infection. Scale bars; 14 μm. **G** Representative images and quantification of ASC-positive cells (magenta) in the hippocampus of 3-month-old 5xFAD mice seven days after HSV-GFP infection. Original magnification × 40, scale bars; 20 μm. Zoom-in images with a scale bar equal to 10 μm. (*n* = 5 mice per group). Data are presented as means ± SEM. *p ≤ 0.05, **p ≤ 0.01, ***p ≤ 0.001, and ****p ≤ 0.0001

Our data suggested that depletion of microglia did not allow to establish a precise role for Aβ deposition and HSV-1 infection in AD pathogenesis. Neuroinflammation, an important hallmark of AD, is associated with Aβ deposition and pathogen invasion [47–49]. To further define the regulation mechanism of HSV-1 and Aβ, we characterized the innate immune signaling pathways induced by HSV-1. Recent reports support the hypothesis that cGAS-STING pathway or NLRP3 inflammasome signaling may act as a driver of neuroinflammation in the brain [50, 51]. To investigate whether the cGAS-STING pathway or the NLRP3 inflammasome pathway is involved in HSV-1-mediated AD pathology, we injected HSV-1 virus into the brains of 5xFAD mice for the indicated time periods and found that HSV-1 infection promotes Aβ accumulation at day 3 and day 7 (Fig. 8A and B), consistent with our findings in Fig. 1.

Next, we examined the protein markers of signaling cascades of NLRP3 inflammasome and the cGAS-STING pathway in the hippocampus of 5xFAD mice for the indicated time periods. We observed that phosphorylation levels of TBK1 and STING were upregulated in the early stages (6 h–24 h) following HSV-1 infection (Fig. 8C and D). Additionally, NLRP3 and cleaved-caspase1 protein expression levels were upregulated on day 3 and day 7 after HSV-1 infection (Fig. 8C and D). Further immunostaining assay confirmed that the NLRP3 inflammasome was activated in the hippocampus of 3-month-old 5xFAD mice at day 7 after HSV-1 infection (Fig. 8E). This phenomenon was also observed in 1-month-old 5xFAD mice and 6-month-old 5xFAD mice at day 7 after HSV-1 infection (Figure S6). Subsequently, IL-1β production was also assessed by ELISA assay in 1-, 3-, and 6-month-old 5xFAD mice infected with PBS or HSV-GFP. We observed increased IL-1β levels in 1-, 3-, and 6-month-old 5xFAD mice after HSV-1 infection (Figure S6C, S6E and S6H).

We then performed immunofluorescence double staining of NLRP3 along with the microglia marker Iba1, astrocyte marker GFAP, and observed that NLRP3 is mostly expressed in microglia and to a lesser extent in GFP-positive neuronal cells (Fig. 8F and Figure S7A-B). In addition, we detected significantly more ASC specks in 5xFAD mice after HSV-1 infection (Fig. 8G), primarily expressed in microglia (Figure S7C) and responding to Aβ accumulation after HSV-1 infection (Figure S8). These results indicate that the expression pattern of the NLRP3 inflammasome is consistent with Aβ accumulation. Together, our data show that the NLRP3 inflammasome is activated after HSV-1 infection, and its expression trend is consistent with Aβ accumulation.

Intracranial HSV-1 infection disrupts the blood–brain barrier (BBB) and leads to the infiltration of periphery immune cells [52, 53]. Macrophages and T cells infiltrate and play a variety of key roles in the immune defense system in neurodegenerative diseases, CNS injury, and CNS infections. Activated microglia, macrophages, and T cells secrete a series of cytokines (e.g., IL-6, IL-1β, TNFα, and IL-10) and chemokines (e.g., CCL5, CCL2, and MCP-1) to exert different functions [54–58]. To investigate the inflammatory responses after HSV-1 intracranial infection, we performed immunostaining assays using anti-CD3 and anti-F4/80 antibodies along with anti-Iba1 antibody and observed a significant infiltration of T cells and macrophages (Figure S9A-D). Subsequently, we isolated total RNA from the non-infected and HSV-infected hippocampi of 3-month-old 5xFAD mice three or seven days after HSV-1 infection. The results showed that inflammatory genes significantly increased three days after HSV-1 infection (Figure S9E-F). Overall, our data reveal that macrophages and T cells infiltrate and secrete inflammatory factors after HSV-1 infection in 5xFAD mice.

### Blockade of NLRP3 inflammasome signaling reduces Aβ deposition and ameliorates cognitive deficits in 5xFAD mice after HSV-1 infection

**Fig. 9:**
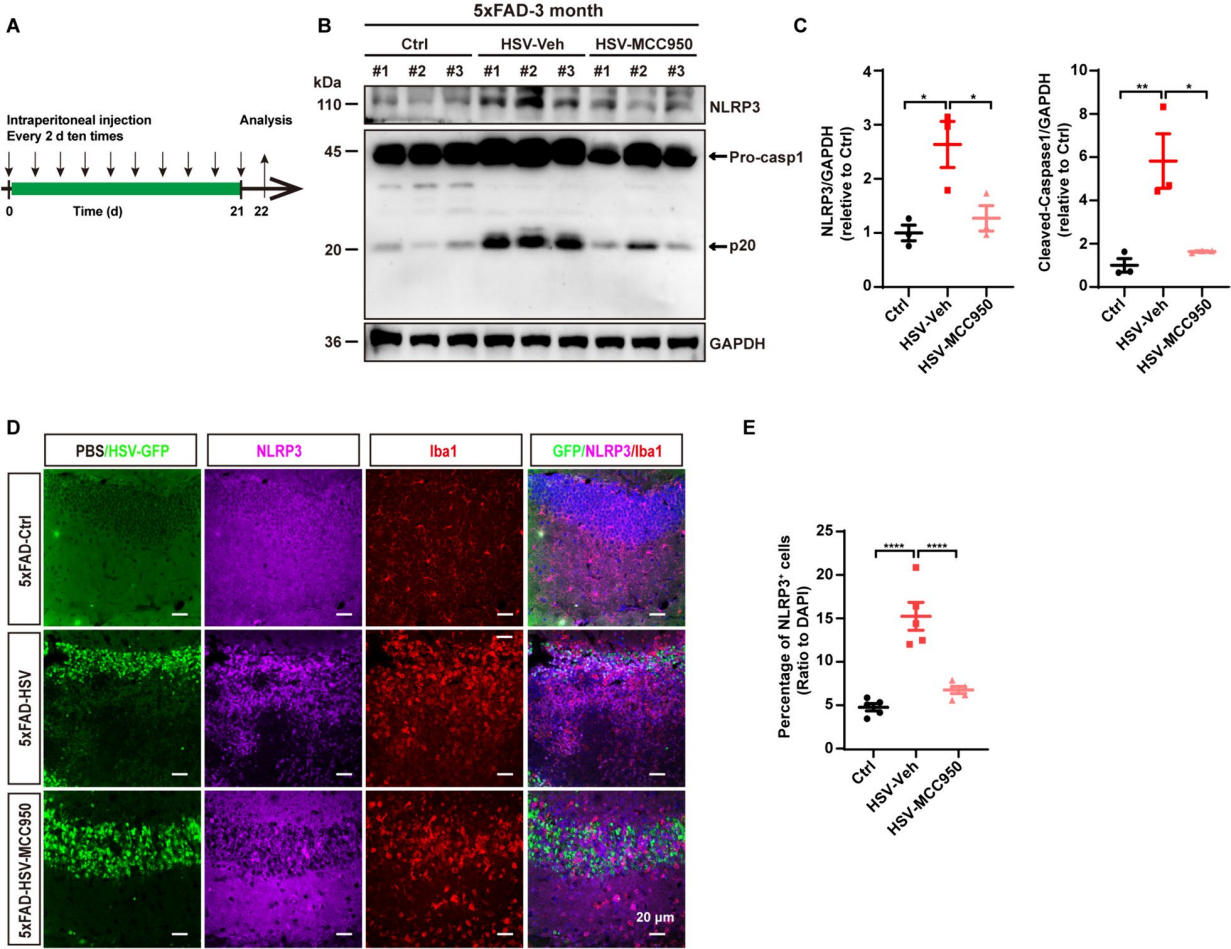
MCC950 treatment inhibits NLRP3 inflammasome activation after HSV-1 infection in 5xFAD mice. **A** Timeline illustrating the intraperitoneal injection of MCC950 into 3-month-old 5xFAD mice following HSV-GFP infection. **B** Western blot analysis of NLRP3 inflammasome protein markers from the hippocampi 21 days after vehicle or MCC950 injection into the 5xFAD mice following HSV-GFP infection. **C** Quantification of the band intensity in **B** using ImageJ software. (*n* = 3 mice per group). **D** Representative images of NLRP3 (magenta) co-stained with Iba1 (red) in the hippocampus of 3-month-old 5xFAD mice treated with MCC950 for 21 days post HSV-GFP infection. Scale bars, 20 μm. **E** Quantification of NLRP3-positive cells in **D** using ImageJ software. (*n* = 5 mice per group). Data are presented as means ± SEM. *p ≤ 0.05, **p ≤ 0.01, and ****p ≤ 0.0001

**Fig. 10:**
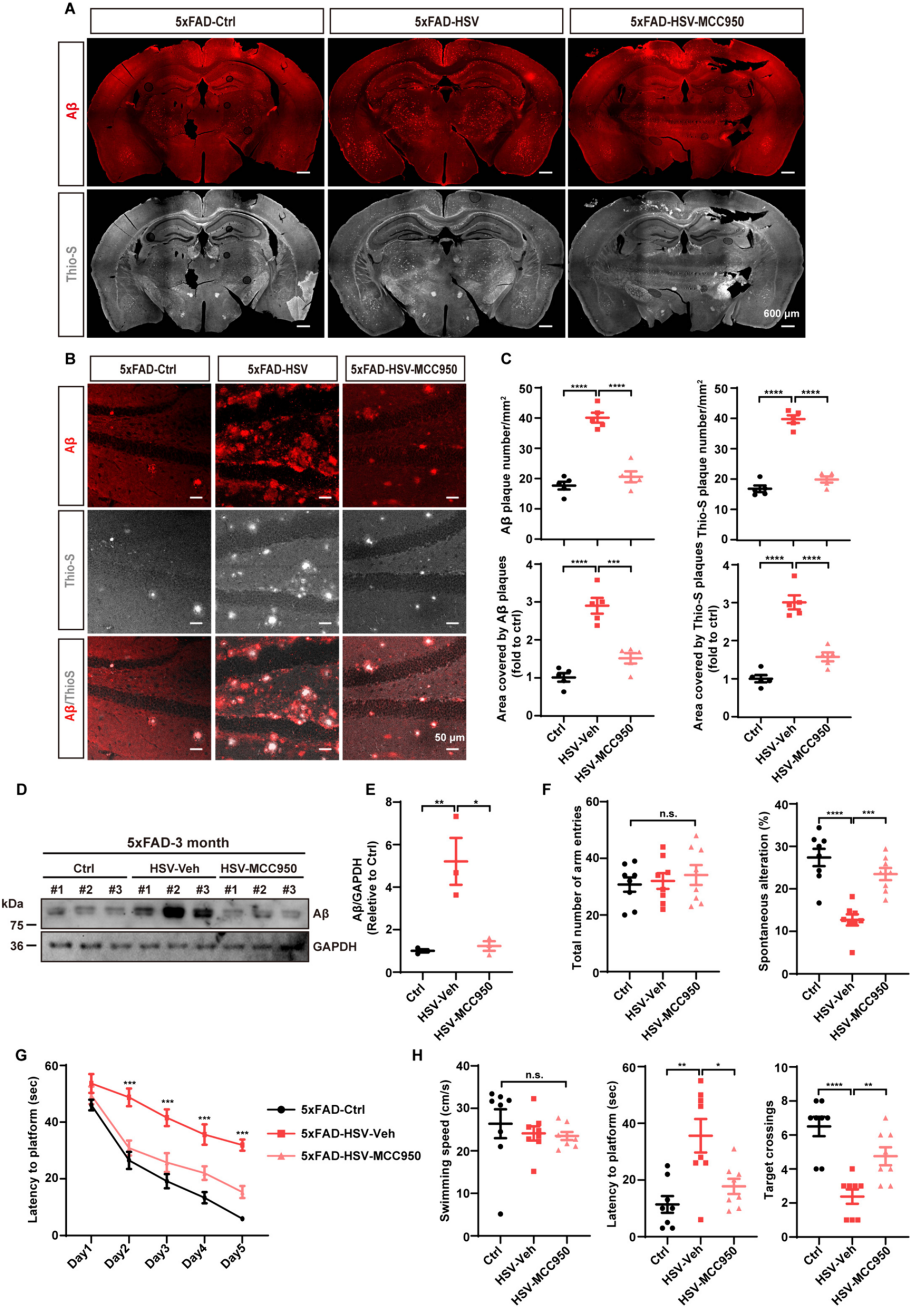
Blockade of the NLRP3 inflammasome signaling reduces Aβ plaque deposition and ameliorates cognitive deficits after HSV-1 infection. **A** Three-month-old 5xFAD mice were administrated with either MCC950 or vehicle for 21 days following HSV-GFP infection. Coronal sections were stained with Aβ (red) and Thio-S (gray). Representative images of the whole brain are shown. Scale bars; 600 μm. **B** Representative images of Aβ (red) and Thio-S (gray) staining in the hippocampus of 3-month-old 5xFAD mice treated with MCC950 for 21 days post HSV-GFP infection. Scale bars, 50 μm. **C** Quantification of Aβ plaques and Aβ area in **A** using ImageJ software. (*n* = 5 mice per group). **D** and **E** Three-month-old 5xFAD mice were administrated with MCC950 for 21 days following HSV-GFP infection. Subsequently, immunoblotting and quantification of Aβ protein levels from the hippocampi are presented. (*n* = 3 mice per group). **F** Three-month-old 5xFAD mice were randomly divided into three groups: control group, HSV-GFP group (vehicle) and HSV-GFP + MCC950 group. Y-maze test was performed to measure the total number of arm entries and spontaneous alteration (%) at 21 days post MCC950 injections. (*n* = 8 mice per group). **G** and **H** Morris water maze was performed to evaluate the spatial learning and memory of 5xFAD mice treated with vehicle or MCC950 following HSV-GFP infection. During the training phase, each group showed improved latency to the platform, but MCC950 treatment significantly improved the latency to the platform compared to the HSV-Veh group (**G**). In the probe test, although MCC950 did not significantly change swimming speed, it ameliorated the impaired latency to the platform and increased the number of target crossings in 5xFAD mice after HSV-GFP infection (**H**). (*n* = 8 mice per group). Data are presented as means ± SEM. n.s.: *p* > 0.05, *p ≤ 0.05, **p ≤ 0.01, ***p ≤ 0.001, and ****p ≤ 0.0001

Our data support a positive correlation between NLRP3 inflammasome activation and Aβ accumulation following HSV-1 infection. To further define if the NLRP3 inflammasome signaling drives Aβ accumulation and AD pathology after HSV-1 infection, we administrated MCC950 sodium, a selective small-molecular inhibitor of the NLRP3 inflammasome, to inhibit inflammasome activation [59, 60]. 5xFAD mice received MCC950 sodium via intraperitoneal injection every second day for 21 days after HSV-1 infection (Fig. 9A). First, we examined the expression levels of the NLRP3 inflammasome through western blot and immunostaining assay and observed a significant inhibition of NLRP3 inflammasome activity after MCC950 treatment following HSV-1 infection (Fig. 9B–E). Next, we evaluated the effects of MCC950 treatment on Aβ deposition. We found that MCC950 treatment significantly reduced the total Aβ burden in 5xFAD mice after HSV-1 infection (Fig. 10A–C). We further examined the Aβ expression amounts by immunoblotting assay. Consistent with the decreased Aβ deposition in imaging data, the expression amounts of Aβ were also decreased in 5xFAD mice treated with MCC950 after HSV-1 infection (Fig. 10D and E). To further investigate whether inflammasome inhibition could affect uptake of HSV-1-infected cells as well as Aβ, we performed an immunostaining assay using an anti-CD68 antibody along with an anti-Aβ antibody to visualize the colocalization of Aβ, HSV-GFP and CD68. Consistent with our data in Fig. 5, microglia preferentially phagocytize HSV-GFP-positive cells after HSV-1 infection, but not Aβ plaques. However, MCC950 treatment did not affect the phagocytic preference of microglia for Aβ or HSV-GFP-positive cells (Figure S10). Overall, inhibition of NLRP3 inflammasome activity did not alter the phagocytic preference of microglia but reduced Aβ deposition.

Then, we assessed the social learning and memory of mice through Y-maze and Morris water-maze tests. Compared with 5xFAD control mice, 5xFAD-HSV mice showed a reduction in spontaneous alterations in the Y-maze test and were less efficient at finding the hidden platform (Fig. 10F–H), indicating a deficit in social learning and memory after HSV-1 infection. However, MCC950 treatment significantly reversed these deficits in 5xFAD mice after HSV-1 infection (Fig. 10F–H). The improvement of these phenotype upon MCC950 treatment was not affected by the total number of arm entries (Fig. 10F) and the swimming speed of mice (Fig. 10H). Collectively, these results suggest that MCC950 treatment to inhibit NLRP3 signaling can ameliorate AD pathology, including Aβ deposition and impaired cognitive function.

## Discussion

**Fig. 11:**
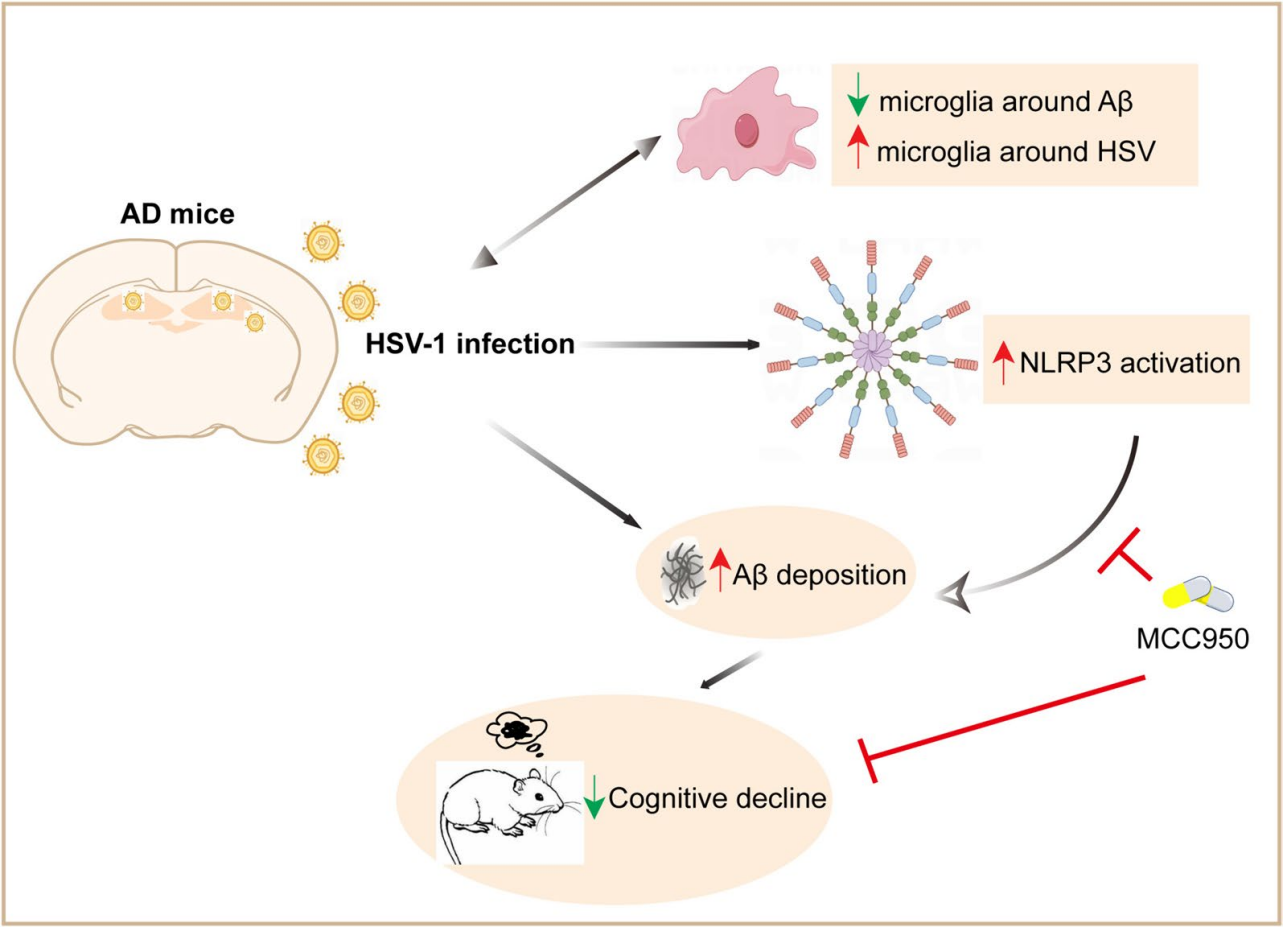
Schematic of HSV-1 infection accelerates the progression of Alzheimer’s disease by modulating microglial phagocytosis and activating NLRP3 pathway

AD is the most common neurodegenerative disorder in the world, and HSV-1 is one of the most common DNA viruses globally. In this study, we characterized the role and regulation mechanism of HSV-1 in 5xFAD mice. Microglia are essential in the modulatory network of HSV-1 infection and Aβ deposition. In addition, we combined viral infection, immunology, biochemistry, cell imaging, and animal approaches to confirm for the first time that HSV-1 infection induced the activation of NLRP3 inflammasome signaling to drive Aβ deposition and AD pathology (Fig. 11). Our results suggest that NLRP3 inflammasome may drive the development of AD in the HSV-1 infection condition, and inhibition of NLRP3 signaling have potential to prevent Aβ accumulation and cognitive decline of AD.

Despite considerable efforts, elucidating the pathogenesis of AD is challenging due to its complex and undefined etiology. Growing evidence implicates that viral or microbial infection of the central nervous system (CNS) are potential pathological factors in the etiology of sporadic AD. The AD pathogen hypothesis suggests that various pathogens act as triggers, initiating a pathological cascade that leads to the accumulation of Aβ [11–14]. According to this hypothesis, Aβ peptides have been shown to possess antimicrobial and antiviral effects and are produced by the CNS as a defense mechanism. Viral or microbial infection result in rapid seeding and accelerated β-amyloid deposition [13]. In this process, these pathogens also activate glial cells and induce a pro-inflammatory innate immune response, ultimately leading to progressive neurodegeneration and dementia [61]. However, the precise mechanism between viral infections and AD remains unclear. In this study, we utilized the approach introduced by Eimer and coauthors [13]. We administrated HSV-1-GFP into the hippocampus of 1-month-old, 3-month-old and 6-month-old 5xFAD mice and observed the aggregation and deposition of Aβ in the whole brain following HSV-1 infection. Moreover, we also observed a significant accelerated cognitive dysfunction in 3-month-old 5xFAD mice following HSV-1 infection. These results are consistent with the previous reports in HSV-1-induced Aβ deposition and suggest HSV-1 infection leads to Aβ deposition and cognitive deficits in AD mouse model.

Microglia are the resident innate immune cells in the CNS and have been shown to play major roles in internalization and degradation of Aβ [62]. They are the primary phagocytes in the CNS for uptake and proteolytic clearance of both soluble and fibrillary forms of Aβ [63]. Inflammatory responses influence the activation status of microglia and subsequently regulate their ability to uptake and degrade Aβ [63]. HSV-1 brain infection leads to persistent activation of microglia, which results in the upregulated expression of type I interferon (IFN) in a cGAS-STING-dependent manner to exert antiviral function [49, 64]. However, abnormal activation of microglia may also lead to dysregulated microglia function, including phagocytosis and inflammation [42, 65–67]. Here, we observed that microglia were activated after HSV-1 infection. In the viral core, microglia often displayed an activated “rounded” and “amoeboid” morphology characterized by shorter processes, larger cell bodies and reduced microglia volumes. Interestingly, we observed that microglia were recruited to the viral core and engulfed HSV-1-GFP-positive neuronal cells, demonstrated by CD68 and Aβ coimmunostaining. Our results demonstrate that the recruitment of microglia to the viral core increased the phagocytic ability to viruses following HSV-1 infection. In this case, no enough microglia would be recruited to clear Aβ, causing Aβ deposition. Our results indicate that microglia modulate their phagocytic preferences, enhancing their uptake of viruses after HSV-1 infection and reducing Aβ uptake, ultimately leading to Aβ accumulation and deposition. In recent years, with the fast development of techniques, growing research on microglia has remarkably revealed their phagocytic roles in neurodegenerative diseases, including Alzheimer’s disease (AD), Parkinson’s disease (PD), Huntington’s disease (HD), frontotemporal dementia (FTD), and amyotrophic lateral sclerosis (ALS) [37, 68–70]. Microglia have a high phagocytic capacity and can clear pathological protein aggregates (Aβ, α-synuclein, and TDP43) [71]. Nevertheless, excessive extracellular protein aggregate release and autophagy impairment both contribute to pathological progression and neurodegeneration. Thus, enhancing microglial phagocytosis is considered a promising strategy for the therapy of neurodegenerative diseases.

Microglia execute their roles as the first line of defense against CNS infection through their immune response mediated by immune receptors [72, 73], including NLR-mediated responses such as the NLRP3 inflammasome [35, 36], and nucleic acids receptor-mediated responses, such as RIG-1 and cGAS [34, 38, 74]. Previous studies have found that NLRP3 is activated in AD mouse models and AD patients, contributing to the progression of AD pathology. NLRP3 inflammasome deficiency resulted in decreased deposition of Aβ and reduced tau hyperphosphorylation, ultimately delaying the progression of AD pathology [51, 75]. In this study, we observed that the NLRP3 inflammasome signaling was activated on day 3 and 7 after HSV-1 infection, with its activation trend being correlated with Aβ accumulation. Therefore, we speculated that the NLRP3 inflammasome was involved in the accumulation of Aβ. To testify this hypothesis, we employed a selective small-molecular inhibitor (MCC950) of the NLRP3 inflammasome signaling to inhibit inflammasome activation and revealed that MCC950 treatment significantly prevented Aβ deposition and alleviated the cognitive impairments induced by HSV-1 infection. These findings suggested that NLRP3 inflammasome activation mediates HSV-1-induced Aβ pathology in 5xFAD mice. Other selective NLRP3 inflammasome inhibitors, such as OLT1177 (Dapansutrile), exert therapeutic effects in the experimental autoimmune encephalopathy (EAE) mouse model and attenuate the infiltration of CD4 T cells [76]. Stavudine (D4T), stimulates Aβ phagocytosis by macrophages to eliminate Aβ [77]. Overall, previous studies and our work indicate that patients with AD and HSV-1 infection may potentially benefit from NLRP3-targetted treatment strategies.

As a typical DNA virus, HSV-1 could activate cGAS-STING DNA sensing pathway [38, 78]. In addition, previous study reveals that HSV-1 infection could induce releases of mitochondrial DNA (mtDNA) into cytosol to activate cGAS-STING signaling. Indeed, we detected strong activation of cGAS-STING signaling at early stage after HSV-1 infection in this study. Recent studies show that abnormal activation of cGAS-STING signaling pathway is a driven-factor of aging and neurodegeneration [79]. Of note, the aberrant cGAS-STING activation are also involved in AD [80–83]. The mtDNA release into cytosol of the microglial could activate cGAS-STING signaling to contribute to AD pathogenesis in 5xFAD mice [81]. Tau can also activate cGAS-STING pathway by inducing microglia mtDNA leakage [82]. Consistently, cGAS deletion or inhibitors of cGAS (or STING inhibitor) could restrict cGAS-STING pathway and mitigate AD progress in different AD disease models [80, 81]. Therefore, we hypothesize that, in AD, distinct sources of innate immune components can be activated in different time course to promote neuroinflammation. We propose that distinct innate immune signaling pathways, including cGAS-STING pathway and NLRP3 inflammasome, may contribute to neuroinflammatory phenomena and AD pathogenesis with distinct mechanisms. Further studies are required to investigate how these innate immune pathways are regulated and cooperates with each other during the pathological phenomena of AD.

Aβ proteins, herpesviruses, and innate immunity are all ancient (at least 300 million years old). Understanding the underlying regulatory mechanisms in brain and neurodegenerative disorders would be very interesting and valuable. While previous reports have suggested a potential link between HSV-1 and Aβ accumulation through the use of AD mouse model and 3D human brain model [11, 13, 67, 84], the cognitive impairment and the precise modulatory mechanism behind it remain unclear. In this study, we demonstrate that HSV-1 infection leads to Aβ deposition, activation of NLRP3 inflammasome, and accelerated cognitive deficits in 5xFAD mice. Our data reveal that microglia and the NLRP3 inflammasome play crucial role in AD pathology induced by HSV-1 infection. Our results provide some information to unravel the mechanism of HSV-1-induced AD pathogenesis, suggesting new therapeutic approaches for this multifactorial and devastating neurodegenerative disease.

### Supplementary Information

Additional file 1: Figure S1. HSV-1 infection does not alter APP processing. (A and B) Immunoblotting (A) and quantification (B) of APP, PS1, ADAM10 and BACE1 proteins in the hippocampus of 1-month-old 5xFAD mice (Ctrl), 5xFAD mice infected with PBS (Vehicle) or 5xFAD mice seven days after HSV-GFP infection (HSV-GFP). (*n* = 3 mice per group). (C and D) Immunoblotting (C) and quantification (D) of APP, PS1, ADAM10 and BACE1 proteins in the hippocampus of 3-month-old 5xFAD mice (Ctrl), 5xFAD mice infected with PBS (Vehicle) or 5xFAD mice seven days after HSV-GFP infection (HSV-GFP). (*n* = 3 mice per group). (E and F) Immunoblotting (E) and quantification (F) of APP, PS1, ADAM10 and BACE1 proteins in the hippocampus of 6-month-old 5xFAD mice (Ctrl), 5xFAD mice infected with PBS (Vehicle) or 5xFAD mice seven days after HSV-GFP infection (HSV-GFP). (*n* = 3 mice per group). Data are presented as means ± SEM. n.s.: *p* > 0.05.

Additional file 2: Figure S2. Enhanced microglial phagocytosis induced by HSV-1 infection in 5xFAD mice. (A) Representative images and quantification of CD68-positive cells (magenta) in the hippocampus of 1-month-old 5xFAD mice seven days after HSV-GFP infection. Original magnification × 40, scale bars; 20 μm. Zoom-in images with a scale bar equal to 10 μm. (*n* = 5 mice per group). (B) Representative images and quantification of CD68-positive cells (magenta) in the hippocampus of 6-month-old 5xFAD mice seven days after HSV-GFP infection. Original magnification × 40, scale bars; 20 μm. Zoom-in images with a scale bar equal to 10 μm. (*n* = 5 mice per group). (C) Representative images of phagocytic microglia (CD68, magenta) co-stained with Aβ (red) in the hippocampus of 3-month-old 5xFAD mice seven days after HSV-GFP infection. Scale bars; 10 μm.

Additional file 3: Figure S3. HSV-1 infection induces enhanced phagocytic of HSV-1-positive cell debris in primary microglia. (A) Representative FACS dot plots showing engulfed GFP-positive cell fragments or 555-labeled Aβ in primary microglia. (B) Quantification of engulfed GFP-positive cell fragments or 555-labeled Aβ in (A). (*n* = 3 mice per group). (C) Representative confocal images of microglial phagocytosis of GFP-positive cell fragments or 555-labeled Aβ after 4 h uptake in cultured primary microglia. Scale bars, 10 μm. (D) Quantification of internalized Aβ using ImageJ software. (*n* = 10 per group). Data are presented as means ± SEM. * *p* ≤ 0.05, ** *p* ≤ 0.01, **** *p* ≤ 0.0001.

Additional file 4: Figure S4. Microglia depletion attenuates microglia activation and phagocytosis. (A) Representative images of phagocytic microglia (CD68, magenta) co-stained with Iba1 (red) in the hippocampus of 1-month-old 5xFAD mice seven days after HSV-GFP infection following 21 days treatment with PLX3397. Scale bars, 50 μm. (B) Quantification of phagocytic microglia (CD68^+^ cells) in (A) using ImageJ software. (*n* = 5 mice per group). (C) Representative images of phagocytic microglia (CD68, magenta) co-stained with Iba1 (red) in the hippocampus of 6-month-old 5xFAD mice seven days after HSV-GFP infection following 21 days treatment with PLX3397. Scale bars, 50 μm. (D) Quantification of phagocytic microglia (CD68^+^ cells or Iba1^+^ cells) in (C) using ImageJ software. (*n* = 5 mice per group). Data are presented as means ± SEM. ****: *p* ≤ 0.0001.

Additional file 5: Figure S5. Microglia depletion increases Aβ plaque deposition in 6-month-old 5xFAD mice after HSV-1 infection. (A) Representative images of Aβ plaques and plaque area in the whole brain of 6-month-old 5xFAD mice seven days after HSV-GFP infection following 21 days treatment with PLX3397. Scale bars, 200 μm. (B) Representative images of Aβ plaques (red) in the hippocampus of 6-month-old 5xFAD mice seven days after HSV-GFP infection following 21 days treatment with PLX3397. Scale bars, 50 μm. (C) Quantification of Aβ plaques and plaque area in the whole brain of 6-month-old 5xFAD mice seven days after HSV-GFP infection following 21 days treatment with PLX3397. (*n* = 5 mice per group). Data are presented as means ± SEM. *: *p* ≤ 0.05.

Additional file 6: Figure S6. The NLRP3 inflammasome is activated in 1-month-old and 6-month-old 5xFAD mice following HSV-1 infection. (A and B) Immunoblotting (A) and quantification (B) of NLRP3 inflammasome protein markers from the hippocampi of 1-month-old 5xFAD mice seven days after HSV-GFP infection. (*n* = 3 mice per group). (C) The expression of IL-1β is determined by ELISA assay in 1-month-old 5xFAD mice seven days after HSV-GFP infection. (*n* = 3 mice per group). (D) Representative images of NLRP3 (magenta) co-stained with Iba1 (red) and quantification of NLRP3-positive cells in the hippocampus of 1-month-old 5xFAD mice seven days after HSV-GFP infection. Scale bars, 20 μm. (*n* = 5 mice per group). (E) The expression of IL-1β is determined by ELISA assay in 3-month-old 5xFAD mice seven days after HSV-GFP infection. (*n* = 3 mice per group). (F and G) Immunoblotting (F) and quantification (G) of NLRP3 inflammasome protein markers from the hippocampi of 6-month-old 5xFAD mice seven days after HSV-GFP infection. (*n* = 3 mice per group). (H) The expression of IL-1β is determined by ELISA assay in 6-month-old 5xFAD mice seven days after HSV-GFP infection. (*n* = 3 mice per group). (I) Representative images of NLRP3 (magenta) co-stained with Iba1 (red) and quantification of NLRP3-positive cells in the hippocampus of 6-month-old 5xFAD mice seven days after HSV-GFP infection. Scale bars, 20 μm. (*n* = 5 mice per group). Data are presented as means ± SEM. n.s.: *p* > 0.05, ***: *p* ≤ 0.001, ****: *p* ≤ 0.0001.

Additional file 7: Figure S7. NLRP3 is mostly expressed in microglia in 5xFAD mice following HSV-1 infection. (A) Representative images of microglia (Iba1, red) co-stained with NLRP3 (magenta) in the hippocampus of 3-month-old 5xFAD mice seven days after HSV-GFP infection. Scale bars, 20 μm. (B) Representative images of NLRP3 (magenta) co-stained with GFP in the hippocampus of 3-month-old 5xFAD mice seven days after HSV-GFP infection. Original magnification × 40, scale bars; 20 μm. Zoom-in images with a scale bar equal to 10 μm, 5 μm. (C) Representative images of microglia (Iba1, red) co-stained with ASC (magenta) in the hippocampus of 3-month-old 5xFAD mice seven days after HSV-GFP infection. Scale bars, 20 μm.

Additional file 8: Figure S8. ASC specks responding to Aβ accumulation in 5xFAD mice following HSV-1 infection. (A) Representative images of ASC (magenta) co-stained with Aβ (red) in the hippocampus of 3-month-old 5xFAD mice seven days after HSV-GFP infection. Original magnification × 40, scale bars; 20 μm. Zoom-in images with a scale bar equal to 10 μm. (*n* = 4 mice per group). (B) Representative images of ASC (magenta) co-stained with Aβ (red) in the hippocampus of 6-month-old 5xFAD mice seven days after HSV-GFP infection. Original magnification × 40, scale bars; 20 μm. Zoom-in images with a scale bar equal to 10 μm. (*n* = 4 mice per group).

Additional file 9: Figure S9. HSV-1 infection induces T cell and macrophage infiltration and dramatically induces inflammatory response. (A) Representative images of microglia (Iba1, red) co-stained with T cells (CD3, magenta) in the hippocampus of 3-month-old 5xFAD mice three days after HSV-GFP infection. Scale bars, 50 μm. (B) Quantification of CD3^+^ cells in the hippocampus of 3-month-old 5xFAD mice infected with PBS or HSV-GFP (1 × 10^5^ PFUs/hippocampus). (*n* = 4 mice per group). (C) Representative images of microglia (Iba1, red) co-stained with macrophages cells (F4/80, magenta) in the hippocampus of 3-month-old 5xFAD mice three days after HSV-GFP infection. Scale bars, 50 μm. (D) Quantification of F4/80^+^ cells in the hippocampus of 3-month-old 5xFAD mice infected with PBS or HSV-GFP (1 × 10^5^ PFUs/hippocampus). (*n* = 4 mice per group). (E and F) HSV-GFP viruses were administered into 3-month-old 5xFAD mice. Hippocampi from non-infected and HSV-infected mice were collected three and seven days post-infection and homogenized for gene expression analysis by real-time PCR. (*n* = 3 mice per group). Data are presented as means ± SEM. * *p* ≤ 0.05, ** *p* ≤ 0.01, *** *p* ≤ 0.001, **** *p* ≤ 0.0001.

Additional file 10: Figure S10. MCC950 treatment does not affect the phagocytic preference of microglia following HSV-1 infection in 5xFAD mice. (A) Representative images of phagocytic microglia (CD68, blue) co-stained with Aβ plaques (magenta) in the hippocampus of 5xFAD mice treated with MCC950 for 21 days following HSV-1 infection. Three-dimensional reconstructed enlarged images of dashed-white or dashed-yellow frames show engulfed Aβ plaques or GFP-positive cells in the microglia. Scale bars, 5 μm. (B) Quantification of engulfed Aβ plaques or GFP-positive cells in (A). (*n* = 4 mice per group). Data are presented as means ± SEM. ****p ≤ 0.0001.

## Data Availability

No datasets were generated or analysed during the current study.

## References

[1] Selkoe DJ, Hardy J. The amyloid hypothesis of Alzheimer’s disease at 25 years. EMBO Mol Med. 2016;8:595–608. 10.15252/emmm.201606210. 27025652 10.15252/emmm.201606210 PMC4888851

[2] Li QX, Villemagne VL, Doecke JD, Rembach A, Sarros S, Varghese S, McGlade A, Laughton KM, Pertile KK, Fowler CJ, et al. Alzheimer’s disease normative cerebrospinal fluid biomarkers validated in PET amyloid-beta characterized subjects from the Australian imaging, biomarkers and lifestyle (AIBL) study. J Alzheimers Dis. 2015;48:175–87. 10.3233/JAD-150247. 26401938 10.3233/JAD-150247

[3] Bertram L, Lill CM, Tanzi RE. The genetics of Alzheimer disease: back to the future. Neuron. 2010;68:270–81. 10.1016/j.neuron.2010.10.013. 20955934 10.1016/j.neuron.2010.10.013

[4] Griciuc A, Serrano-Pozo A, Parrado AR, Lesinski AN, Asselin CN, Mullin K, Hooli B, Choi SH, Hyman BT, Tanzi RE. Alzheimer’s disease risk gene CD33 inhibits microglial uptake of amyloid beta. Neuron. 2013;78:631–43. 10.1016/j.neuron.2013.04.014. 23623698 10.1016/j.neuron.2013.04.014 PMC3706457

[5] Haass C, Selkoe DJ. Soluble protein oligomers in neurodegeneration: lessons from the Alzheimer’s amyloid beta-peptide. Nat Rev Mol Cell Biol. 2007;8:101–12. 10.1038/nrm2101. 17245412 10.1038/nrm2101

[6] Querfurth HW, LaFerla FM. Alzheimer’s disease. N Engl J Med. 2010;362:329–44. 10.1056/NEJMra0909142. 20107219 10.1056/NEJMra0909142

[7] Tanzi RE, Bertram L. Twenty years of the Alzheimer’s disease amyloid hypothesis: a genetic perspective. Cell. 2005;120:545–55. 10.1016/j.cell.2005.02.008. 15734686 10.1016/j.cell.2005.02.008

[8] Hardy J, Selkoe DJ. The amyloid hypothesis of Alzheimer’s disease: progress and problems on the road to therapeutics. Science. 2002;297:353–6. 10.1126/science.1072994. 12130773 10.1126/science.1072994

[9] Miklossy J. Emerging roles of pathogens in Alzheimer disease. Expert Rev Mol Med. 2011;13: e30. 10.1017/S1462399411002006. 21933454 10.1017/S1462399411002006

[10] Itzhaki RF, Lathe R, Balin BJ, Ball MJ, Bearer EL, Braak H, Bullido MJ, Carter C, Clerici M, Cosby SL, et al. Microbes and Alzheimer’s disease. J Alzheimers Dis. 2016;51:979–84. 10.3233/JAD-160152. 26967229 10.3233/JAD-160152 PMC5457904

[11] Cairns DM, Rouleau N, Parker RN, Walsh KG, Gehrke L, Kaplan DL. A 3D human brain-like tissue model of herpes-induced Alzheimer’s disease. Sci Adv. 2020;6:eaay8828. 10.1126/sciadv.aay8828. 32494701 10.1126/sciadv.aay8828 PMC7202879

[12] Itzhaki RF. Herpes and Alzheimer’s disease: subversion in the central nervous system and how it might be halted. J Alzheimers Dis. 2016;54:1273–81. 10.3233/JAD-160607. 27497484 10.3233/JAD-160607

[13] Eimer WA, Vijaya Kumar DK, Navalpur Shanmugam NK, Rodriguez AS, Mitchell T, Washicosky KJ, Gyorgy B, Breakefield XO, Tanzi RE, Moir RD. Alzheimer’s disease-associated beta-amyloid is rapidly seeded by herpesviridae to protect against brain infection. Neuron. 2018;100:1527–32. 10.1016/j.neuron.2018.11.043. 30571943 10.1016/j.neuron.2018.11.043

[14] Kumar DK, Choi SH, Washicosky KJ, Eimer WA, Tucker S, Ghofrani J, Lefkowitz A, McColl G, Goldstein LE, Tanzi RE, Moir RD. Amyloid-beta peptide protects against microbial infection in mouse and worm models of Alzheimer’s disease. Sci Transl Med. 2016. 10.1126/scitranslmed.aaf1059. 27225182 10.1126/scitranslmed.aaf1059 PMC5505565

[15] Balin BJ, Little CS, Hammond CJ, Appelt DM, Whittum-Hudson JA, Gerard HC, Hudson AP. Chlamydophila pneumoniae and the etiology of late-onset Alzheimer’s disease. J Alzheimers Dis. 2008;13:371–80. 10.3233/jad-2008-13403. 18487846 10.3233/jad-2008-13403

[16] Miklossy J. Alzheimer’s disease—a neurospirochetosis. Analysis of the evidence following Koch’s and Hill’s criteria. J Neuroinflammation. 2011;8:90. 10.1186/1742-2094-8-90. 21816039 10.1186/1742-2094-8-90 PMC3171359

[17] Pisa D, Alonso R, Juarranz A, Rabano A, Carrasco L. Direct visualization of fungal infection in brains from patients with Alzheimer’s disease. J Alzheimers Dis. 2015;43:613–24. 10.3233/JAD-141386. 25125470 10.3233/JAD-141386

[18] Piacentini R, De Chiara G, Li Puma DD, Ripoli C, Marcocci ME, Garaci E, Palamara AT, Grassi C. HSV-1 and Alzheimer’s disease: more than a hypothesis. Front Pharmacol. 2014;5:97. 10.3389/fphar.2014.00097. 24847267 10.3389/fphar.2014.00097 PMC4019841

[19] De Chiara G, Piacentini R, Fabiani M, Mastrodonato A, Marcocci ME, Limongi D, Napoletani G, Protto V, Coluccio P, Celestino I, et al. Recurrent herpes simplex virus-1 infection induces hallmarks of neurodegeneration and cognitive deficits in mice. PLoS Pathog. 2019;15: e1007617. 10.1371/journal.ppat.1007617. 30870531 10.1371/journal.ppat.1007617 PMC6417650

[20] Itzhaki RF. Corroboration of a major role for herpes simplex virus type 1 in Alzheimer’s disease. Front Aging Neurosci. 2018;10:324. 10.3389/fnagi.2018.00324. 30405395 10.3389/fnagi.2018.00324 PMC6202583

[21] Duarte LF, Farias MA, Alvarez DM, Bueno SM, Riedel CA, Gonzalez PA. Herpes simplex virus type 1 infection of the central nervous system: insights into proposed interrelationships with neurodegenerative disorders. Front Cell Neurosci. 2019;13:46. 10.3389/fncel.2019.00046. 30863282 10.3389/fncel.2019.00046 PMC6399123

[22] Readhead B, Haure-Mirande JV, Funk CC, Richards MA, Shannon P, Haroutunian V, Sano M, Liang WS, Beckmann ND, Price ND, et al. Multiscale analysis of independent Alzheimer’s cohorts finds disruption of molecular, genetic, and clinical networks by human herpesvirus. Neuron. 2018;99(64–82): e67. 10.1016/j.neuron.2018.05.023. 10.1016/j.neuron.2018.05.023 PMC6551233 29937276

[23] Jamieson GA, Maitland NJ, Wilcock GK, Craske J, Itzhaki RF. Latent herpes simplex virus type 1 in normal and Alzheimer’s disease brains. J Med Virol. 1991;33:224–7. 10.1002/jmv.1890330403. 1649907 10.1002/jmv.1890330403

[24] Jamieson GA, Maitland NJ, Wilcock GK, Yates CM, Itzhaki RF. Herpes simplex virus type 1 DNA is present in specific regions of brain from aged people with and without senile dementia of the Alzheimer type. J Pathol. 1992;167:365–8. 10.1002/path.1711670403. 1328575 10.1002/path.1711670403

[25] Gu H, Roizman B. Herpes simplex virus-infected cell protein 0 blocks the silencing of viral DNA by dissociating histone deacetylases from the CoREST-REST complex. Proc Natl Acad Sci U S A. 2007;104:17134–9. 10.1073/pnas.0707266104. 17939992 10.1073/pnas.0707266104 PMC2040395

[26] Marques CP, Hu S, Sheng W, Lokensgard JR. Microglial cells initiate vigorous yet non-protective immune responses during HSV-1 brain infection. Virus Res. 2006;121:1–10. 10.1016/j.virusres.2006.03.009. 16621100 10.1016/j.virusres.2006.03.009

[27] Graeber MB. Changing face of microglia. Science. 2010;330:783–8. 10.1126/science.1190929. 21051630 10.1126/science.1190929

[28] Nimmerjahn A, Kirchhoff F, Helmchen F. Resting microglial cells are highly dynamic surveillants of brain parenchyma in vivo. Science. 2005;308:1314–8. 10.1126/science.1110647. 15831717 10.1126/science.1110647

[29] Parkhurst CN, Yang G, Ninan I, Savas JN, Yates JR 3rd, Lafaille JJ, Hempstead BL, Littman DR, Gan WB. Microglia promote learning-dependent synapse formation through brain-derived neurotrophic factor. Cell. 2013;155:1596–609. 10.1016/j.cell.2013.11.030. 24360280 10.1016/j.cell.2013.11.030 PMC4033691

[30] Ransohoff RM. How neuroinflammation contributes to neurodegeneration. Science. 2016;353:777–83. 10.1126/science.aag2590. 27540165 10.1126/science.aag2590

[31] Ransohoff RM. Neuroinflammation: surprises from the sanitary engineers. Nature. 2016;532:185–6. 10.1038/nature17881. 27049948 10.1038/nature17881

[32] Van Eldik LJ, Carrillo MC, Cole PE, Feuerbach D, Greenberg BD, Hendrix JA, Kennedy M, Kozauer N, Margolin RA, Molinuevo JL, et al. The roles of inflammation and immune mechanisms in Alzheimer’s disease. Alzheimers Dement (N Y). 2016;2:99–109. 10.1016/j.trci.2016.05.001. 29067297 10.1016/j.trci.2016.05.001 PMC5644267

[33] Furr SR, Chauhan VS, Moerdyk-Schauwecker MJ, Marriott I. A role for DNA-dependent activator of interferon regulatory factor in the recognition of herpes simplex virus type 1 by glial cells. J Neuroinflammation. 2011;8:99. 10.1186/1742-2094-8-99. 21838860 10.1186/1742-2094-8-99 PMC3168419

[34] Reinert LS, Lopusna K, Winther H, Sun C, Thomsen MK, Nandakumar R, Mogensen TH, Meyer M, Vaegter C, Nyengaard JR, et al. Sensing of HSV-1 by the cGAS-STING pathway in microglia orchestrates antiviral defence in the CNS. Nat Commun. 2016;7:13348. 10.1038/ncomms13348. 27830700 10.1038/ncomms13348 PMC5109551

[35] Jacobs SR, Damania B. NLRs, inflammasomes, and viral infection. J Leukoc Biol. 2012;92:469–77. 10.1189/jlb.0312132. 22581934 10.1189/jlb.0312132 PMC4046246

[36] Johnson KE, Chikoti L, Chandran B. Herpes simplex virus 1 infection induces activation and subsequent inhibition of the IFI16 and NLRP3 inflammasomes. J Virol. 2013;87:5005–18. 10.1128/JVI.00082-13. 23427152 10.1128/JVI.00082-13 PMC3624293

[37] Paolicelli RC, Jawaid A, Henstridge CM, Valeri A, Merlini M, Robinson JL, Lee EB, Rose J, Appel S, Lee VM, et al. TDP-43 depletion in microglia promotes amyloid clearance but also induces synapse loss. Neuron. 2017;95(297–308): e296. 10.1016/j.neuron.2017.05.037. 10.1016/j.neuron.2017.05.037 PMC5519492 28669544

[38] Li XD, Wu J, Gao D, Wang H, Sun L, Chen ZJ. Pivotal roles of cGAS-cGAMP signaling in antiviral defense and immune adjuvant effects. Science. 2013;341:1390–4. 10.1126/science.1244040. 23989956 10.1126/science.1244040 PMC3863637

[39] Wei X, Zhang L, Yang Y, Hou Y, Xu Y, Wang Z, Su H, Han F, Han J, Liu P, et al. LL-37 transports immunoreactive cGAMP to activate STING signaling and enhance interferon-mediated host antiviral immunity. Cell Rep. 2022;39: 110880. 10.1016/j.celrep.2022.110880. 35649354 10.1016/j.celrep.2022.110880

[40] Hou Y, Wang Z, Liu P, Wei X, Zhang Z, Fan S, Zhang L, Han F, Song Y, Chu L, Zhang C. SMPDL3A is a cGAMP-degrading enzyme induced by LXR-mediated lipid metabolism to restrict cGAS-STING DNA sensing. Immunity. 2023;56(2492–2507): e2410. 10.1016/j.immuni.2023.10.001. 10.1016/j.immuni.2023.10.001 37890481

[41] Huang Y, Xu Z, Xiong S, Sun F, Qin G, Hu G, Wang J, Zhao L, Liang YX, Wu T, et al. Repopulated microglia are solely derived from the proliferation of residual microglia after acute depletion. Nat Neurosci. 2018;21:530–40. 10.1038/s41593-018-0090-8. 29472620 10.1038/s41593-018-0090-8

[42] Pascoal TA, Benedet AL, Ashton NJ, Kang MS, Therriault J, Chamoun M, Savard M, Lussier FZ, Tissot C, Karikari TK, et al. Microglial activation and tau propagate jointly across Braak stages. Nat Med. 2021;27:1592–9. 10.1038/s41591-021-01456-w. 34446931 10.1038/s41591-021-01456-w

[43] DeBiasi RL, Kleinschmidt-DeMasters BK, Richardson-Burns S, Tyler KL. Central nervous system apoptosis in human herpes simplex virus and cytomegalovirus encephalitis. J Infect Dis. 2002;186:1547–57. 10.1086/345375. 12447729 10.1086/345375

[44] Liu CC, Zhao N, Yamaguchi Y, Cirrito JR, Kanekiyo T, Holtzman DM, Bu G. Neuronal heparan sulfates promote amyloid pathology by modulating brain amyloid-beta clearance and aggregation in Alzheimer’s disease. Sci Transl Med. 2016. 10.1126/scitranslmed.aad3650. 27030596 10.1126/scitranslmed.aad3650 PMC5512541

[45] Elmore MR, Najafi AR, Koike MA, Dagher NN, Spangenberg EE, Rice RA, Kitazawa M, Matusow B, Nguyen H, West BL, Green KN. Colony-stimulating factor 1 receptor signaling is necessary for microglia viability, unmasking a microglia progenitor cell in the adult brain. Neuron. 2014;82:380–97. 10.1016/j.neuron.2014.02.040. 24742461 10.1016/j.neuron.2014.02.040 PMC4161285

[46] Spangenberg EE, Lee RJ, Najafi AR, Rice RA, Elmore MR, Blurton-Jones M, West BL, Green KN. Eliminating microglia in Alzheimer’s mice prevents neuronal loss without modulating amyloid-beta pathology. Brain. 2016;139:1265–81. 10.1093/brain/aww016. 26921617 10.1093/brain/aww016 PMC5006229

[47] Komine O, Yamanaka K. Neuroinflammation in motor neuron disease. Nagoya J Med Sci. 2015;77:537–49. 26663933 PMC4664586

[48] Streit WJ, Mrak RE, Griffin WS. Microglia and neuroinflammation: a pathological perspective. J Neuroinflammation. 2004;1:14. 10.1186/1742-2094-1-14. 15285801 10.1186/1742-2094-1-14 PMC509427

[49] Paludan SR, Bowie AG, Horan KA, Fitzgerald KA. Recognition of herpesviruses by the innate immune system. Nat Rev Immunol. 2011;11:143–54. 10.1038/nri2937. 21267015 10.1038/nri2937 PMC3686362

[50] Venegas C, Kumar S, Franklin BS, Dierkes T, Brinkschulte R, Tejera D, Vieira-Saecker A, Schwartz S, Santarelli F, Kummer MP, et al. Microglia-derived ASC specks cross-seed amyloid-beta in Alzheimer’s disease. Nature. 2017;552:355–61. 10.1038/nature25158. 29293211 10.1038/nature25158

[51] Ising C, Venegas C, Zhang S, Scheiblich H, Schmidt SV, Vieira-Saecker A, Schwartz S, Albasset S, McManus RM, Tejera D, et al. NLRP3 inflammasome activation drives tau pathology. Nature. 2019;575:669–73. 10.1038/s41586-019-1769-z. 31748742 10.1038/s41586-019-1769-z PMC7324015

[52] Mott K, Brick DJ, van Rooijen N, Ghiasi H. Macrophages are important determinants of acute ocular HSV-1 infection in immunized mice. Invest Ophthalmol Vis Sci. 2007;48:5605–15. 10.1167/iovs.07-0894. 18055810 10.1167/iovs.07-0894

[53] Chan WL, Javanovic T, Lukic ML. Infiltration of immune T cells in the brain of mice with herpes simplex virus-induced encephalitis. J Neuroimmunol. 1989;23:195–201. 10.1016/0165-5728(89)90051-9. 2787806 10.1016/0165-5728(89)90051-9 PMC7119877

[54] Esiri MM, Drummond CW, Morris CS. Macrophages and microglia in HSV-1 infected mouse brain. J Neuroimmunol. 1995;62:201–5. 10.1016/0165-5728(95)00123-8. 7499509 10.1016/0165-5728(95)00123-8

[55] Tumpey TM, Cheng H, Cook DN, Smithies O, Oakes JE, Lausch RN. Absence of macrophage inflammatory protein-1alpha prevents the development of blinding herpes stromal keratitis. J Virol. 1998;72:3705–10. 10.1128/JVI.72.5.3705-3710.1998. 9557652 10.1128/JVI.72.5.3705-3710.1998 PMC109592

[56] Tumpey TM, Cheng H, Yan XT, Oakes JE, Lausch RN. Chemokine synthesis in the HSV-1-infected cornea and its suppression by interleukin-10. J Leukoc Biol. 1998;63:486–92. 10.1002/jlb.63.4.486. 9544579 10.1002/jlb.63.4.486

[57] Ghiasi H, Hofman FM, Cai S, Perng GC, Nesburn AB, Wechsler SL. Vaccination with different HSV-1 glycoproteins induces different patterns of ocular cytokine responses following HSV-1 challenge of vaccinated mice. Vaccine. 1999;17:2576–82. 10.1016/s0264-410x(99)00056-0. 10418905 10.1016/s0264-410x(99)00056-0

[58] Chan SH, Perussia B, Gupta JW, Kobayashi M, Pospisil M, Young HA, Wolf SF, Young D, Clark SC, Trinchieri G. Induction of interferon gamma production by natural killer cell stimulatory factor: characterization of the responder cells and synergy with other inducers. J Exp Med. 1991;173:869–79. 10.1084/jem.173.4.869. 1672545 10.1084/jem.173.4.869 PMC2190821

[59] Coll RC, Robertson AA, Chae JJ, Higgins SC, Munoz-Planillo R, Inserra MC, Vetter I, Dungan LS, Monks BG, Stutz A, et al. A small-molecule inhibitor of the NLRP3 inflammasome for the treatment of inflammatory diseases. Nat Med. 2015;21:248–55. 10.1038/nm.3806. 25686105 10.1038/nm.3806 PMC4392179

[60] Coll RC, Hill JR, Day CJ, Zamoshnikova A, Boucher D, Massey NL, Chitty JL, Fraser JA, Jennings MP, Robertson AAB, Schroder K. MCC950 directly targets the NLRP3 ATP-hydrolysis motif for inflammasome inhibition. Nat Chem Biol. 2019;15:556–9. 10.1038/s41589-019-0277-7. 31086327 10.1038/s41589-019-0277-7

[61] Bates KA, Fonte J, Robertson TA, Martins RN, Harvey AR. Chronic gliosis triggers Alzheimer’s disease-like processing of amyloid precursor protein. Neuroscience. 2002;113:785–96. 10.1016/s0306-4522(02)00230-0. 12182886 10.1016/s0306-4522(02)00230-0

[62] Davalos D, Grutzendler J, Yang G, Kim JV, Zuo Y, Jung S, Littman DR, Dustin ML, Gan WB. ATP mediates rapid microglial response to local brain injury in vivo. Nat Neurosci. 2005;8:752–8. 10.1038/nn1472. 15895084 10.1038/nn1472

[63] Lee CY, Landreth GE. The role of microglia in amyloid clearance from the AD brain. J Neural Transm (Vienna). 2010;117:949–60. 10.1007/s00702-010-0433-4. 20552234 10.1007/s00702-010-0433-4 PMC3653296

[64] McNab F, Mayer-Barber K, Sher A, Wack A, O’Garra A. Type I interferons in infectious disease. Nat Rev Immunol. 2015;15:87–103. 10.1038/nri3787. 25614319 10.1038/nri3787 PMC7162685

[65] Salminen A, Haapasalo A, Kauppinen A, Kaarniranta K, Soininen H, Hiltunen M. Impaired mitochondrial energy metabolism in Alzheimer’s disease: impact on pathogenesis via disturbed epigenetic regulation of chromatin landscape. Prog Neurobiol. 2015;131:1–20. 10.1016/j.pneurobio.2015.05.001. 26001589 10.1016/j.pneurobio.2015.05.001

[66] Liu PP, Xie Y, Meng XY, Kang JS. History and progress of hypotheses and clinical trials for Alzheimer’s disease. Signal Transduct Target Ther. 2019;4:29. 10.1038/s41392-019-0063-8. 31637009 10.1038/s41392-019-0063-8 PMC6799833

[67] Schemmert S, Schartmann E, Zafiu C, Kass B, Hartwig S, Lehr S, Bannach O, Langen KJ, Shah NJ, Kutzsche J, et al. Abeta oligomer elimination restores cognition in transgenic Alzheimer’s mice with full-blown pathology. Mol Neurobiol. 2019;56:2211–23. 10.1007/s12035-018-1209-3. 30003517 10.1007/s12035-018-1209-3 PMC6394605

[68] Gao C, Jiang J, Tan Y, Chen S. Microglia in neurodegenerative diseases: mechanism and potential therapeutic targets. Signal Transduct Target Ther. 2023;8:359. 10.1038/s41392-023-01588-0. 37735487 10.1038/s41392-023-01588-0 PMC10514343

[69] Spiller KJ, Restrepo CR, Khan T, Dominique MA, Fang TC, Canter RG, Roberts CJ, Miller KR, Ransohoff RM, Trojanowski JQ, Lee VM. Microglia-mediated recovery from ALS-relevant motor neuron degeneration in a mouse model of TDP-43 proteinopathy. Nat Neurosci. 2018;21:329–40. 10.1038/s41593-018-0083-7. 29463850 10.1038/s41593-018-0083-7 PMC5857237

[70] Gerrits E, Brouwer N, Kooistra SM, Woodbury ME, Vermeiren Y, Lambourne M, Mulder J, Kummer M, Moller T, Biber K, et al. Distinct amyloid-beta and tau-associated microglia profiles in Alzheimer’s disease. Acta Neuropathol. 2021;141:681–96. 10.1007/s00401-021-02263-w. 33609158 10.1007/s00401-021-02263-w PMC8043951

[71] Zhang W, Wang T, Pei Z, Miller DS, Wu X, Block ML, Wilson B, Zhang W, Zhou Y, Hong JS, Zhang J. Aggregated alpha-synuclein activates microglia: a process leading to disease progression in Parkinson’s disease. FASEB J. 2005;19:533–42. 10.1096/fj.04-2751com. 15791003 10.1096/fj.04-2751com

[72] Prinz M, Priller J. Microglia and brain macrophages in the molecular age: from origin to neuropsychiatric disease. Nat Rev Neurosci. 2014;15:300–12. 10.1038/nrn3722. 24713688 10.1038/nrn3722

[73] Colonna M, Butovsky O. Microglia function in the central nervous system during health and neurodegeneration. Annu Rev Immunol. 2017;35:441–68. 10.1146/annurev-immunol-051116-052358. 28226226 10.1146/annurev-immunol-051116-052358 PMC8167938

[74] Crill EK, Furr-Rogers SR, Marriott I. RIG-I is required for VSV-induced cytokine production by murine glia and acts in combination with DAI to initiate responses to HSV-1. Glia. 2015;63:2168–80. 10.1002/glia.22883. 26146945 10.1002/glia.22883 PMC4600648

[75] Heneka MT, Kummer MP, Stutz A, Delekate A, Schwartz S, Vieira-Saecker A, Griep A, Axt D, Remus A, Tzeng TC, et al. NLRP3 is activated in Alzheimer’s disease and contributes to pathology in APP/PS1 mice. Nature. 2013;493:674–8. 10.1038/nature11729. 23254930 10.1038/nature11729 PMC3812809

[76] Sanchez-Fernandez A, Skouras DB, Dinarello CA, Lopez-Vales R. OLT1177 (Dapansutrile), a selective NLRP3 inflammasome inhibitor, ameliorates experimental autoimmune encephalomyelitis pathogenesis. Front Immunol. 2019;10:2578. 10.3389/fimmu.2019.02578. 31736980 10.3389/fimmu.2019.02578 PMC6839275

[77] La Rosa F, Saresella M, Marventano I, Piancone F, Ripamonti E, Al-Daghri N, Bazzini C, Zoia CP, Conti E, Ferrarese C, Clerici M. Stavudine reduces NLRP3 inflammasome activation and modulates amyloid-beta autophagy. J Alzheimers Dis. 2019;72:401–12. 10.3233/JAD-181259. 31594217 10.3233/JAD-181259

[78] West AP, Khoury-Hanold W, Staron M, Tal MC, Pineda CM, Lang SM, Bestwick M, Duguay BA, Raimundo N, MacDuff DA, et al. Mitochondrial DNA stress primes the antiviral innate immune response. Nature. 2015;520:553–7. 10.1038/nature14156. 25642965 10.1038/nature14156 PMC4409480

[79] Gulen MF, Samson N, Keller A, Schwabenland M, Liu C, Gluck S, Thacker VV, Favre L, Mangeat B, Kroese LJ, et al. cGAS-STING drives ageing-related inflammation and neurodegeneration. Nature. 2023;620:374–80. 10.1038/s41586-023-06373-1. 37532932 10.1038/s41586-023-06373-1 PMC10412454

[80] Udeochu JC, Amin S, Huang Y, Fan L, Torres ERS, Carling GK, Liu B, McGurran H, Coronas-Samano G, Kauwe G, et al. Tau activation of microglial cGAS-IFN reduces MEF2C-mediated cognitive resilience. Nat Neurosci. 2023;26:737–50. 10.1038/s41593-023-01315-6. 37095396 10.1038/s41593-023-01315-6 PMC10166855

[81] Xie X, Ma G, Li X, Zhao J, Zhao Z, Zeng J. Activation of innate immune cGAS-STING pathway contributes to Alzheimer’s pathogenesis in 5xFAD mice. Nat Aging. 2023;3:202–12. 10.1038/s43587-022-00337-2. 37118112 10.1038/s43587-022-00337-2

[82] Jin M, Shiwaku H, Tanaka H, Obita T, Ohuchi S, Yoshioka Y, Jin X, Kondo K, Fujita K, Homma H, et al. Tau activates microglia via the PQBP1-cGAS-STING pathway to promote brain inflammation. Nat Commun. 2021;12:6565. 10.1038/s41467-021-26851-2. 34782623 10.1038/s41467-021-26851-2 PMC8592984

[83] Naguib S, Torres ER, Lopez-Lee C, Fan L, Bhagwat M, Norman K, Lee SI, Zhu J, Ye P, Wong MY, et al. APOE3-R136S mutation confers resilience against tau pathology via cGAS-STING-IFN inhibition. bioRxiv. 2024. 10.1101/2024.04.25.591140. 38712164 10.1101/2024.04.25.591140 PMC11071490

[84] Wozniak MA, Frost AL, Preston CM, Itzhaki RF. Antivirals reduce the formation of key Alzheimer’s disease molecules in cell cultures acutely infected with herpes simplex virus type 1. PLoS ONE. 2011;6: e25152. 10.1371/journal.pone.0025152. 22003387 10.1371/journal.pone.0025152 PMC3189195

