# Supplementary figures and images for: Herpes simplex virus 1 accelerates the progression of Alzheimer’s disease by modulating microglial phagocytosis and activating NLRP3 pathway
Source: J Neuroinflammation. 2024 Jul 18;21:176. doi: 10.1186/s12974-024-03166-9 (PMC11264637; doi:10.1186/s12974-024-03166-9)

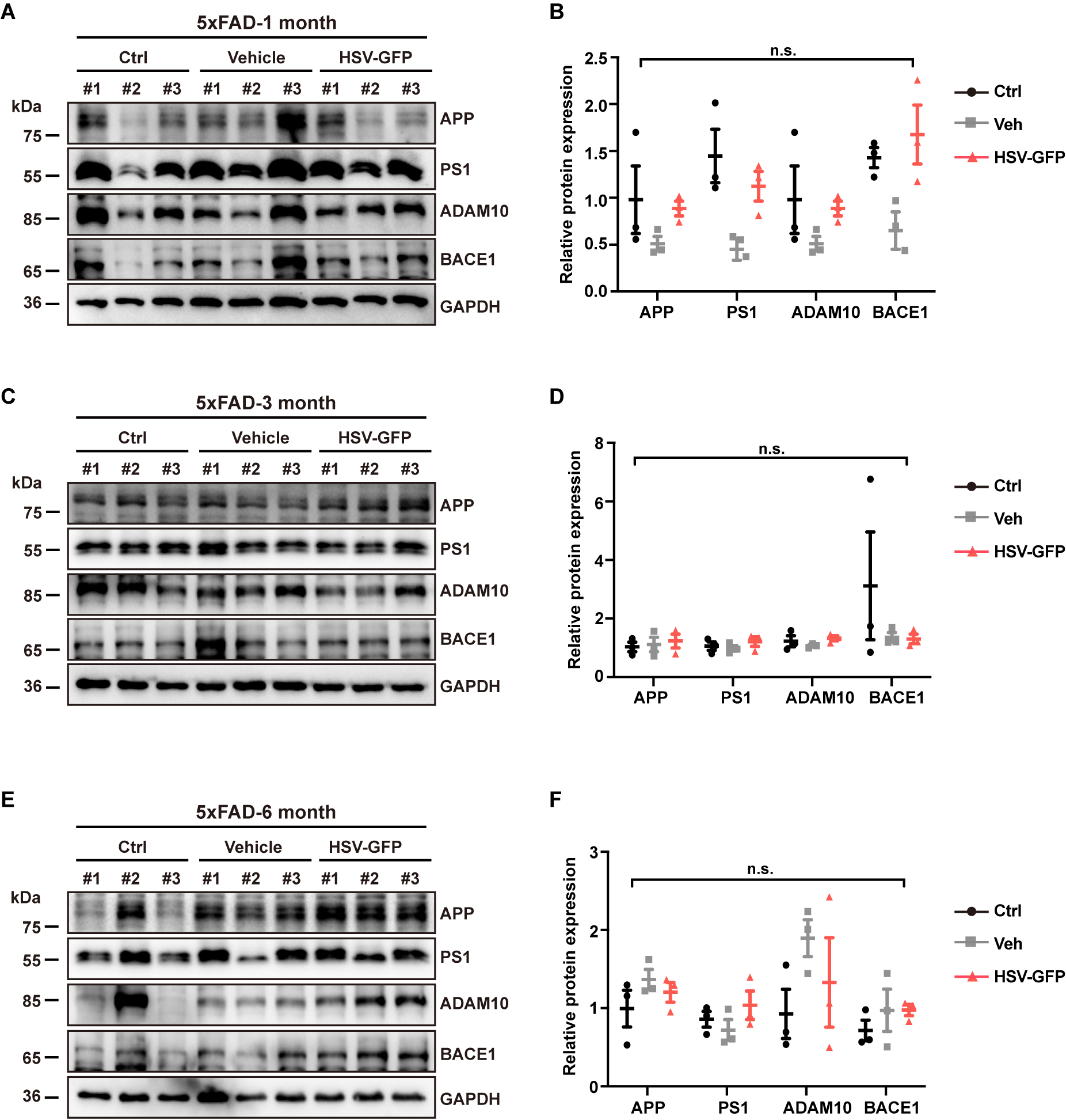

Supplement: Supplementary file 1 — Additional file 1: Figure S1. HSV-1 infection does not alter APP processing. (A and B) Immunoblotting (A) and quantification (B) of APP, PS1, ADAM10 and BACE1 proteins in the hippocampus of 1-month-old 5xFAD mice (Ctrl), 5xFAD mice infected with PBS (Vehicle) or 5xFAD mice seven days after HSV-GFP infection (HSV-GFP). (n = 3 mice per group). (C and D) Immunoblotting (C) and quantification (D) of APP, PS1, ADAM10 and BACE1 proteins in the hippocampus of 3-month-old 5xFAD mice (Ctrl), 5xFAD mice infected with PBS (Vehicle) or 5xFAD mice seven days after HSV-GFP infection (HSV-GFP). (n = 3 mice per group). (E and F) Immunoblotting (E) and quantification (F) of APP, PS1, ADAM10 and BACE1 proteins in the hippocampus of 6-month-old 5xFAD mice (Ctrl), 5xFAD mice infected with PBS (Vehicle) or 5xFAD mice seven days after HSV-GFP infection (HSV-GFP). (n = 3 mice per group). Data are presented as means ± SEM. n.s.: p > 0.05. [file 12974_2024_3166_MOESM1_ESM.tif]

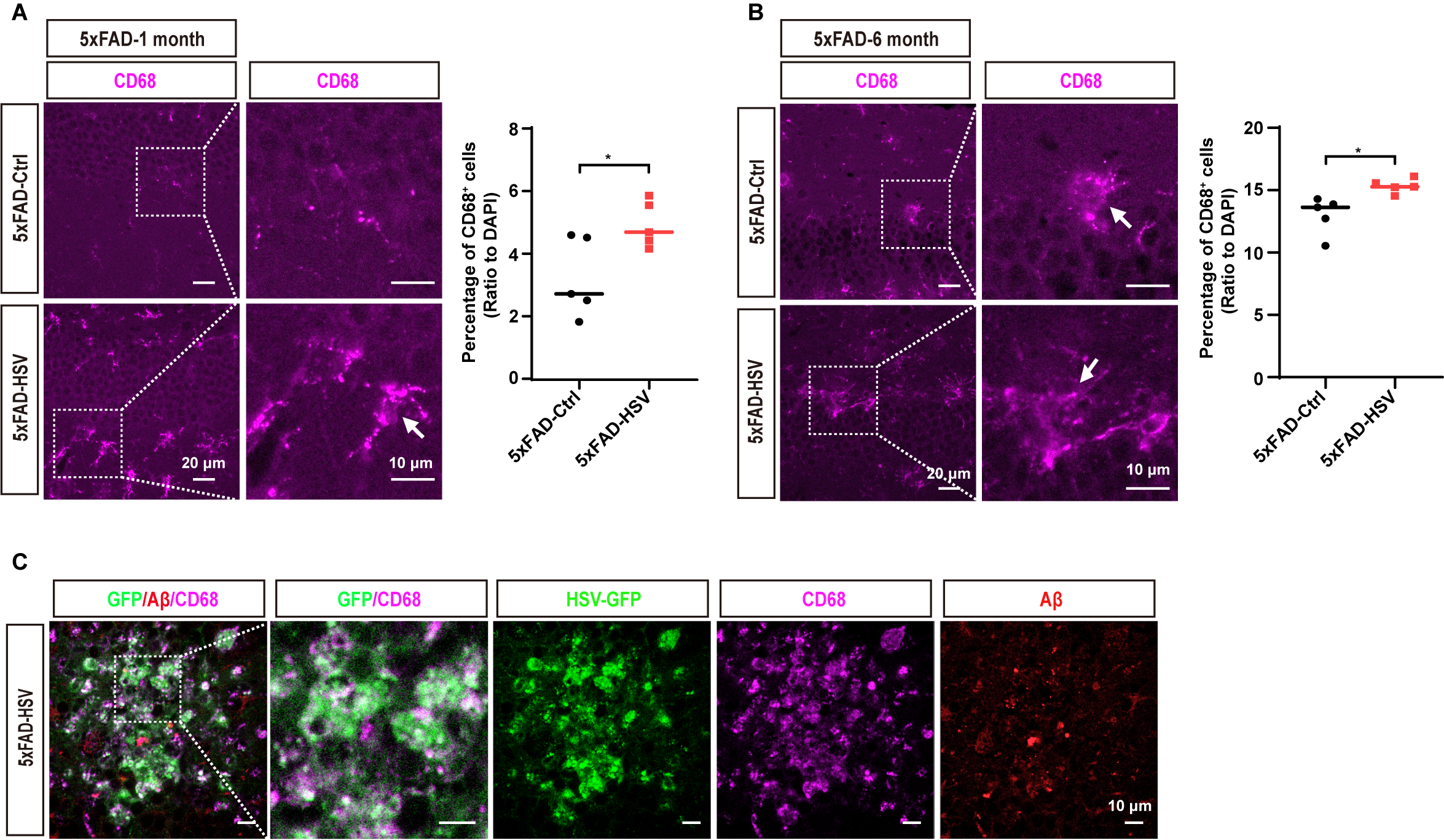

Supplement: Supplementary file 2 — Additional file 2: Figure S2. Enhanced microglial phagocytosis induced by HSV-1 infection in 5xFAD mice. (A) Representative images and quantification of CD68-positive cells (magenta) in the hippocampus of 1-month-old 5xFAD mice seven days after HSV-GFP infection. Original magnification × 40, scale bars; 20 μm. Zoom-in images with a scale bar equal to 10 μm. (n = 5 mice per group). (B) Representative images and quantification of CD68-positive cells (magenta) in the hippocampus of 6-month-old 5xFAD mice seven days after HSV-GFP infection. Original magnification × 40, scale bars; 20 μm. Zoom-in images with a scale bar equal to 10 μm. (n = 5 mice per group). (C) Representative images of phagocytic microglia (CD68, magenta) co-stained with Aβ (red) in the hippocampus of 3-month-old 5xFAD mice seven days after HSV-GFP infection. Scale bars; 10 μm. [file 12974_2024_3166_MOESM2_ESM.tif]

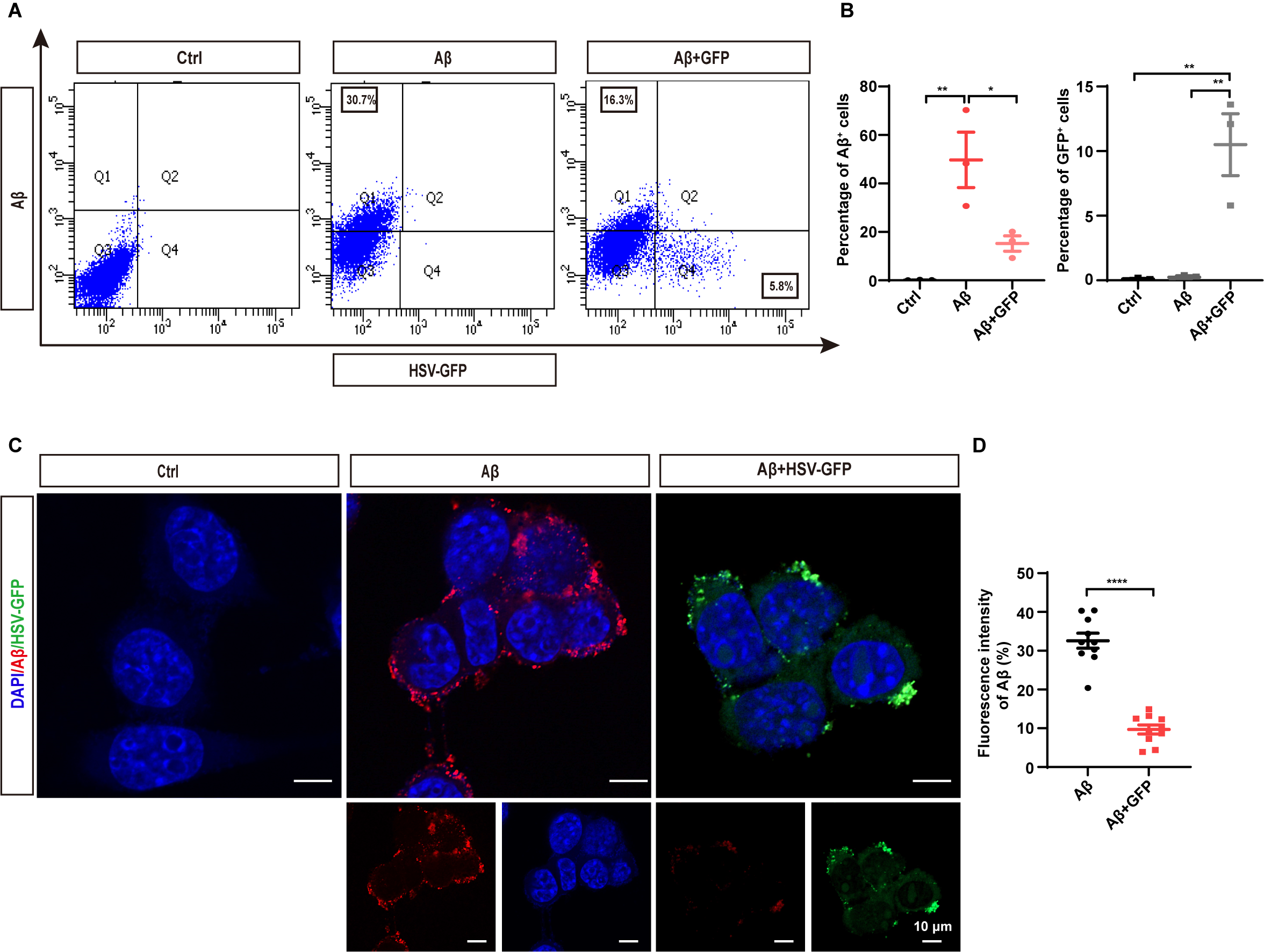

Supplement: Supplementary file 3 — Additional file 3: Figure S3. HSV-1 infection induces enhanced phagocytic of HSV-1-positive cell debris in primary microglia. (A) Representative FACS dot plots showing engulfed GFP-positive cell fragments or 555-labeled Aβ in primary microglia. (B) Quantification of engulfed GFP-positive cell fragments or 555-labeled Aβ in (A). (n = 3 mice per group). (C) Representative confocal images of microglial phagocytosis of GFP-positive cell fragments or 555-labeled Aβ after 4 h uptake in cultured primary microglia. Scale bars, 10 μm. (D) Quantification of internalized Aβ using ImageJ software. (n = 10 per group). Data are presented as means ± SEM. * p ≤ 0.05, ** p ≤ 0.01, **** p ≤ 0.0001. [file 12974_2024_3166_MOESM3_ESM.tif]

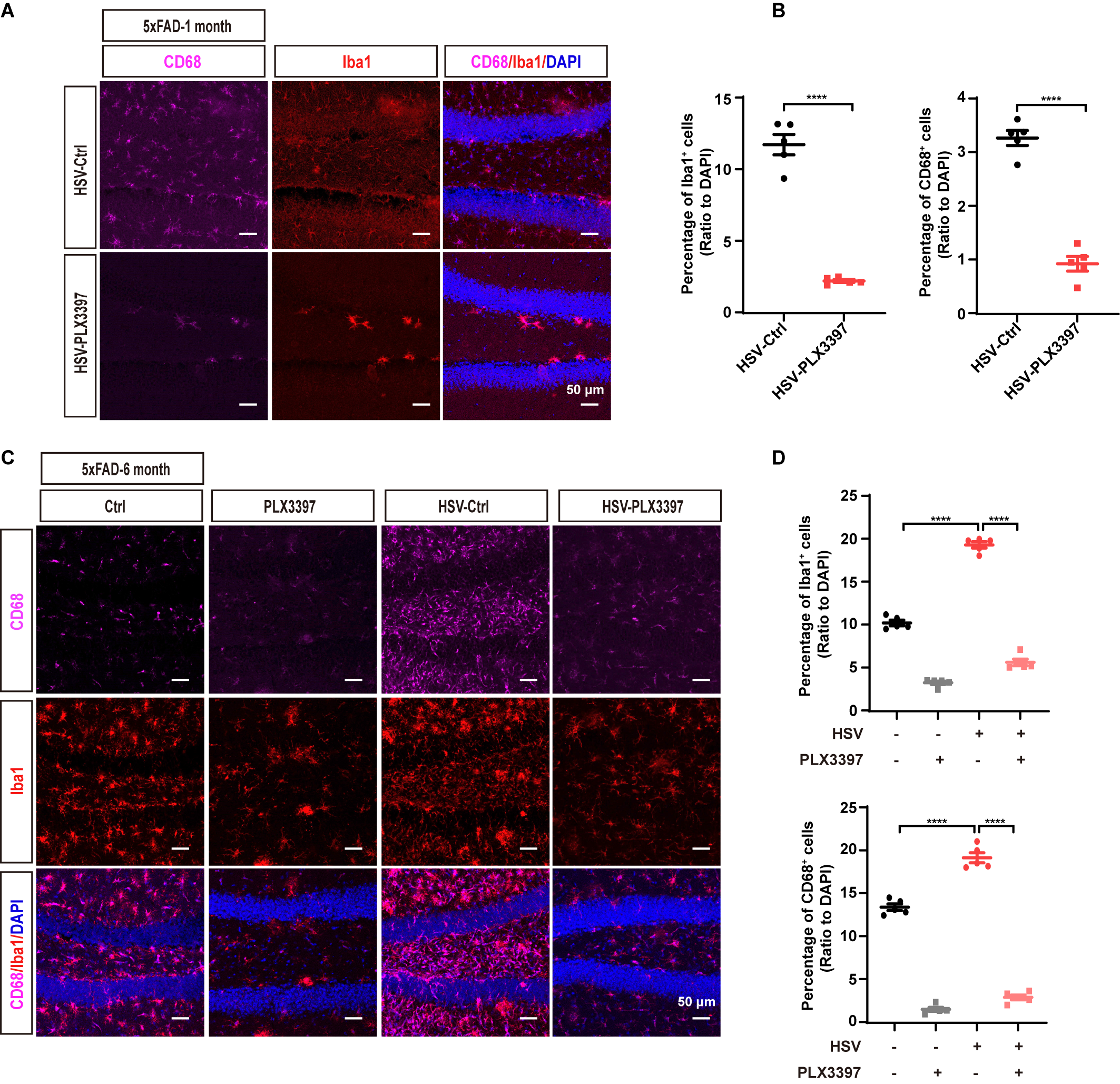

Supplement: Supplementary file 4 — Additional file 4: Figure S4. Microglia depletion attenuates microglia activation and phagocytosis. (A) Representative images of phagocytic microglia (CD68, magenta) co-stained with Iba1 (red) in the hippocampus of 1-month-old 5xFAD mice seven days after HSV-GFP infection following 21 days treatment with PLX3397. Scale bars, 50 μm. (B) Quantification of phagocytic microglia (CD68+ cells) in (A) using ImageJ software. (n = 5 mice per group). (C) Representative images of phagocytic microglia (CD68, magenta) co-stained with Iba1 (red) in the hippocampus of 6-month-old 5xFAD mice seven days after HSV-GFP infection following 21 days treatment with PLX3397. Scale bars, 50 μm. (D) Quantification of phagocytic microglia (CD68+ cells or Iba1+ cells) in (C) using ImageJ software. (n = 5 mice per group). Data are presented as means ± SEM. ****: p ≤ 0.0001. [file 12974_2024_3166_MOESM4_ESM.tif]

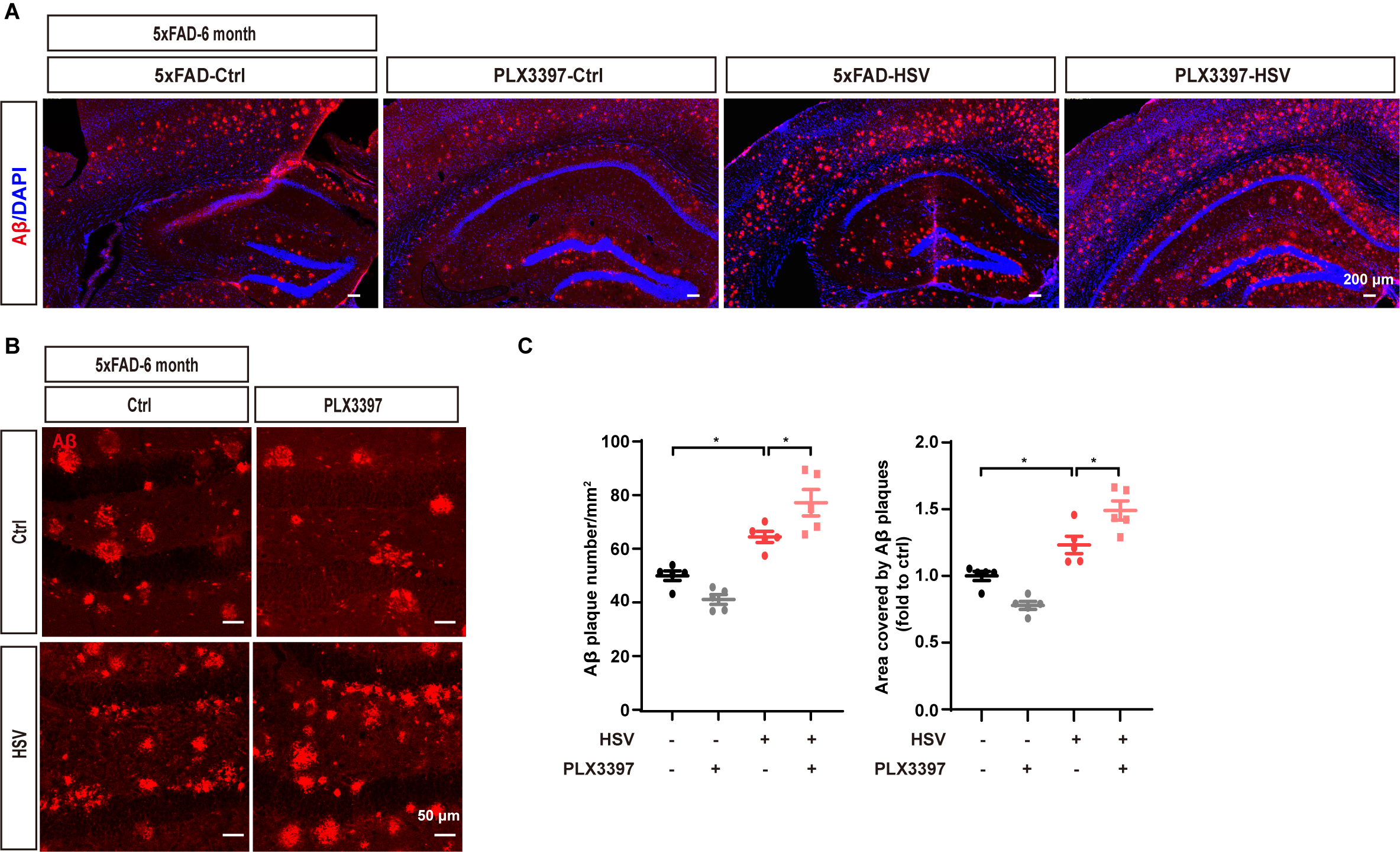

Supplement: Supplementary file 5 — Additional file 5: Figure S5. Microglia depletion increases Aβ plaque deposition in 6-month-old 5xFAD mice after HSV-1 infection. (A) Representative images of Aβ plaques and plaque area in the whole brain of 6-month-old 5xFAD mice seven days after HSV-GFP infection following 21 days treatment with PLX3397. Scale bars, 200 μm. (B) Representative images of Aβ plaques (red) in the hippocampus of 6-month-old 5xFAD mice seven days after HSV-GFP infection following 21 days treatment with PLX3397. Scale bars, 50 μm. (C) Quantification of Aβ plaques and plaque area in the whole brain of 6-month-old 5xFAD mice seven days after HSV-GFP infection following 21 days treatment with PLX3397. (n = 5 mice per group). Data are presented as means ± SEM. *: p ≤ 0.05. [file 12974_2024_3166_MOESM5_ESM.tif]

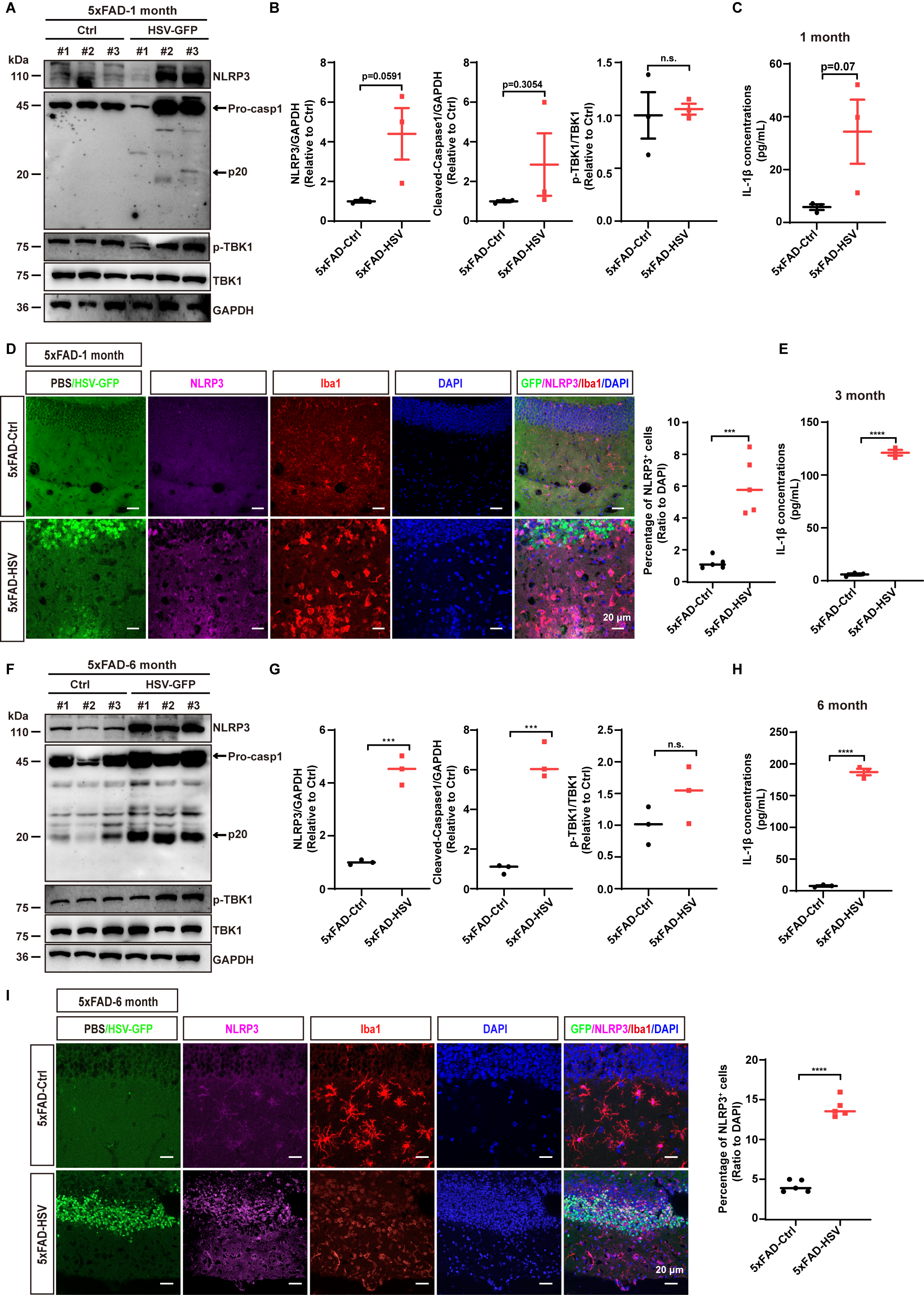

Supplement: Supplementary file 6 — Additional file 6: Figure S6. The NLRP3 inflammasome is activated in 1-month-old and 6-month-old 5xFAD mice following HSV-1 infection. (A and B) Immunoblotting (A) and quantification (B) of NLRP3 inflammasome protein markers from the hippocampi of 1-month-old 5xFAD mice seven days after HSV-GFP infection. (n = 3 mice per group). (C) The expression of IL-1β is determined by ELISA assay in 1-month-old 5xFAD mice seven days after HSV-GFP infection. (n = 3 mice per group). (D) Representative images of NLRP3 (magenta) co-stained with Iba1 (red) and quantification of NLRP3-positive cells in the hippocampus of 1-month-old 5xFAD mice seven days after HSV-GFP infection. Scale bars, 20 μm. (n = 5 mice per group). (E) The expression of IL-1β is determined by ELISA assay in 3-month-old 5xFAD mice seven days after HSV-GFP infection. (n = 3 mice per group). (F and G) Immunoblotting (F) and quantification (G) of NLRP3 inflammasome protein markers from the hippocampi of 6-month-old 5xFAD mice seven days after HSV-GFP infection. (n = 3 mice per group). (H) The expression of IL-1β is determined by ELISA assay in 6-month-old 5xFAD mice seven days after HSV-GFP infection. (n = 3 mice per group). (I) Representative images of NLRP3 (magenta) co-stained with Iba1 (red) and quantification of NLRP3-positive cells in the hippocampus of 6-month-old 5xFAD mice seven days after HSV-GFP infection. Scale bars, 20 μm. (n = 5 mice per group). Data are presented as means ± SEM. n.s.: p > 0.05, ***: p ≤ 0.001, ****: p ≤ 0.0001. [file 12974_2024_3166_MOESM6_ESM.tif]

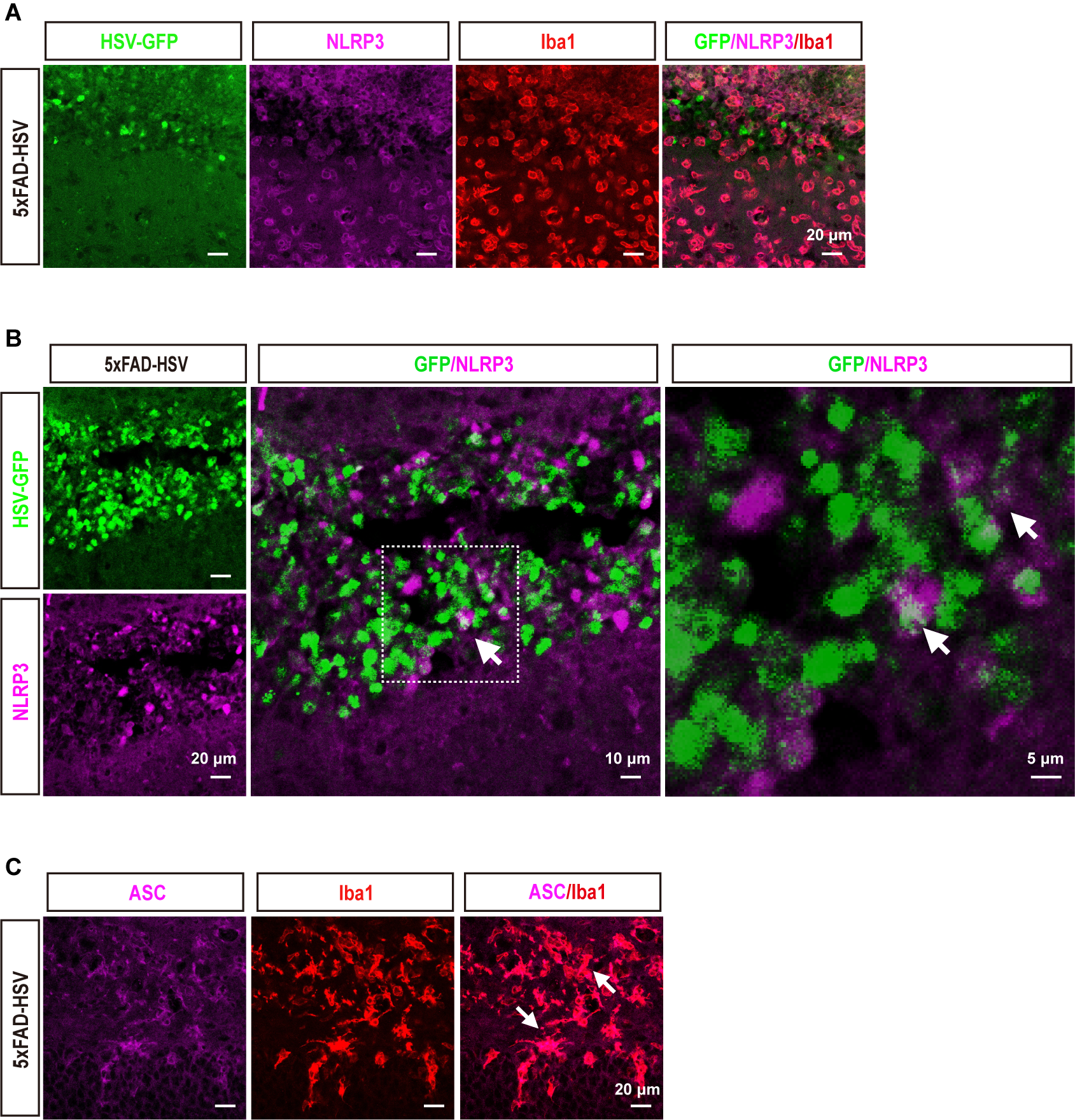

Supplement: Supplementary file 7 — Additional file 7: Figure S7. NLRP3 is mostly expressed in microglia in 5xFAD mice following HSV-1 infection. (A) Representative images of microglia (Iba1, red) co-stained with NLRP3 (magenta) in the hippocampus of 3-month-old 5xFAD mice seven days after HSV-GFP infection. Scale bars, 20 μm. (B) Representative images of NLRP3 (magenta) co-stained with GFP in the hippocampus of 3-month-old 5xFAD mice seven days after HSV-GFP infection. Original magnification × 40, scale bars; 20 μm. Zoom-in images with a scale bar equal to 10 μm, 5 μm. (C) Representative images of microglia (Iba1, red) co-stained with ASC (magenta) in the hippocampus of 3-month-old 5xFAD mice seven days after HSV-GFP infection. Scale bars, 20 μm. [file 12974_2024_3166_MOESM7_ESM.tif]

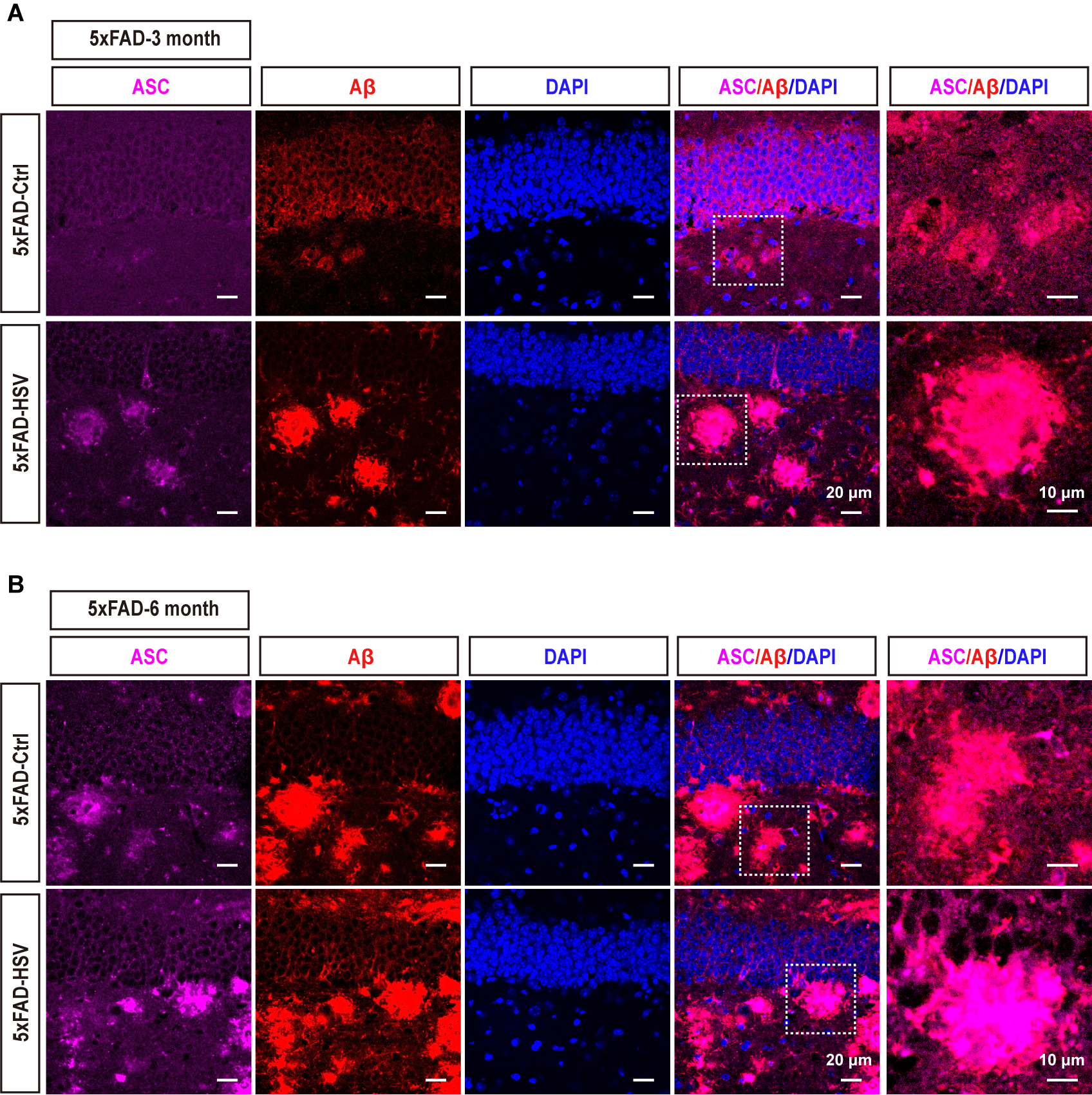

Supplement: Supplementary file 8 — Additional file 8: Figure S8. ASC specks responding to Aβ accumulation in 5xFAD mice following HSV-1 infection. (A) Representative images of ASC (magenta) co-stained with Aβ (red) in the hippocampus of 3-month-old 5xFAD mice seven days after HSV-GFP infection. Original magnification × 40, scale bars; 20 μm. Zoom-in images with a scale bar equal to 10 μm. (n = 4 mice per group). (B) Representative images of ASC (magenta) co-stained with Aβ (red) in the hippocampus of 6-month-old 5xFAD mice seven days after HSV-GFP infection. Original magnification × 40, scale bars; 20 μm. Zoom-in images with a scale bar equal to 10 μm. (n = 4 mice per group). [file 12974_2024_3166_MOESM8_ESM.tif]

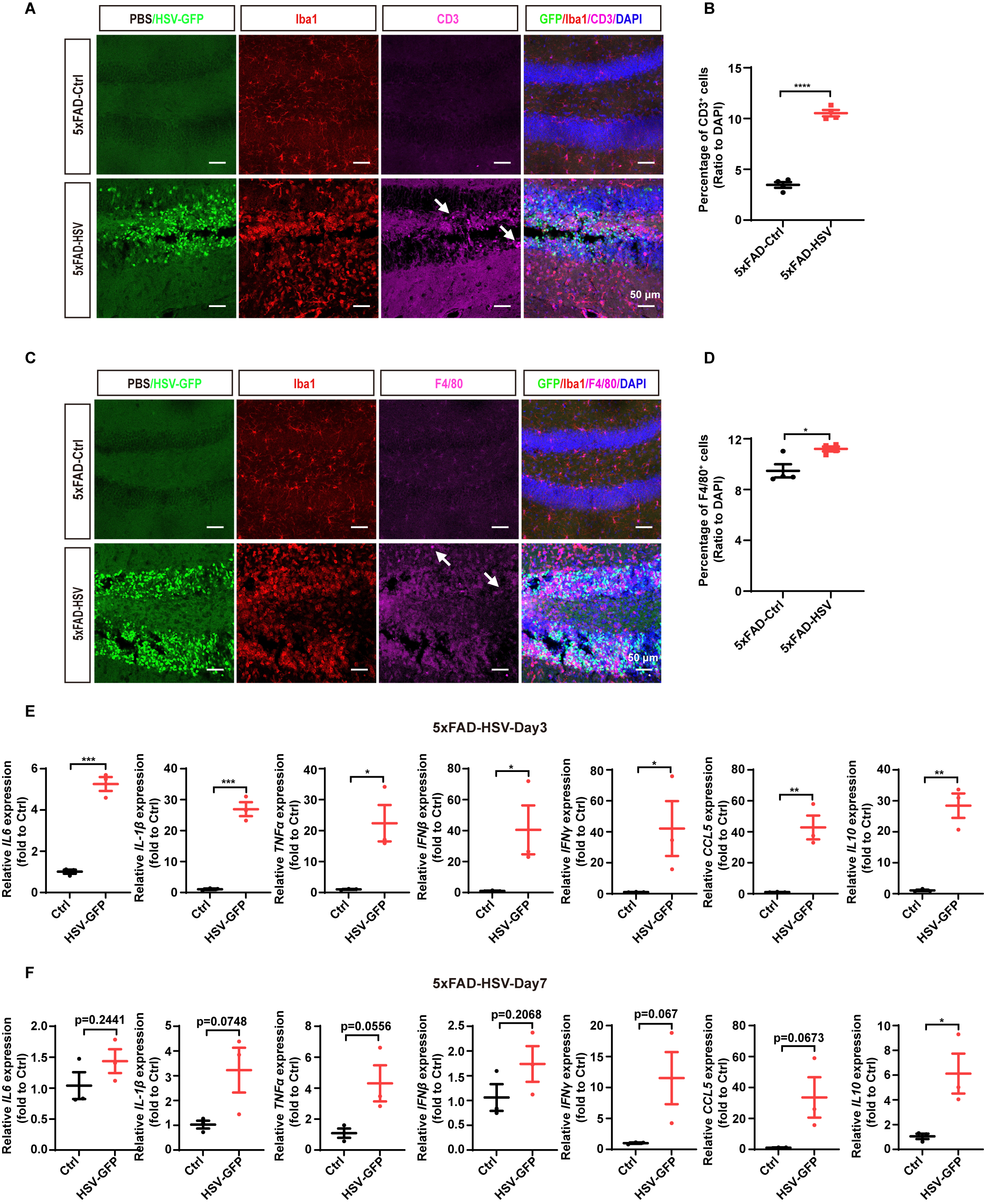

Supplement: Supplementary file 9 — Additional file 9: Figure S9. HSV-1 infection induces T cell and macrophage infiltration and dramatically induces inflammatory response. (A) Representative images of microglia (Iba1, red) co-stained with T cells (CD3, magenta) in the hippocampus of 3-month-old 5xFAD mice three days after HSV-GFP infection. Scale bars, 50 μm. (B) Quantification of CD3+ cells in the hippocampus of 3-month-old 5xFAD mice infected with PBS or HSV-GFP (1 × 10^5^ PFUs/hippocampus). (n = 4 mice per group). (C) Representative images of microglia (Iba1, red) co-stained with macrophages cells (F4/80, magenta) in the hippocampus of 3-month-old 5xFAD mice three days after HSV-GFP infection. Scale bars, 50 μm. (D) Quantification of F4/80+ cells in the hippocampus of 3-month-old 5xFAD mice infected with PBS or HSV-GFP (1 × 10^5^ PFUs/hippocampus). (n = 4 mice per group). (E and F) HSV-GFP viruses were administered into 3-month-old 5xFAD mice. Hippocampi from non-infected and HSV-infected mice were collected three and seven days post-infection and homogenized for gene expression analysis by real-time PCR. (n = 3 mice per group). Data are presented as means ± SEM. * p ≤ 0.05, ** p ≤ 0.01, *** p ≤ 0.001, **** p ≤ 0.0001. [file 12974_2024_3166_MOESM9_ESM.tif]

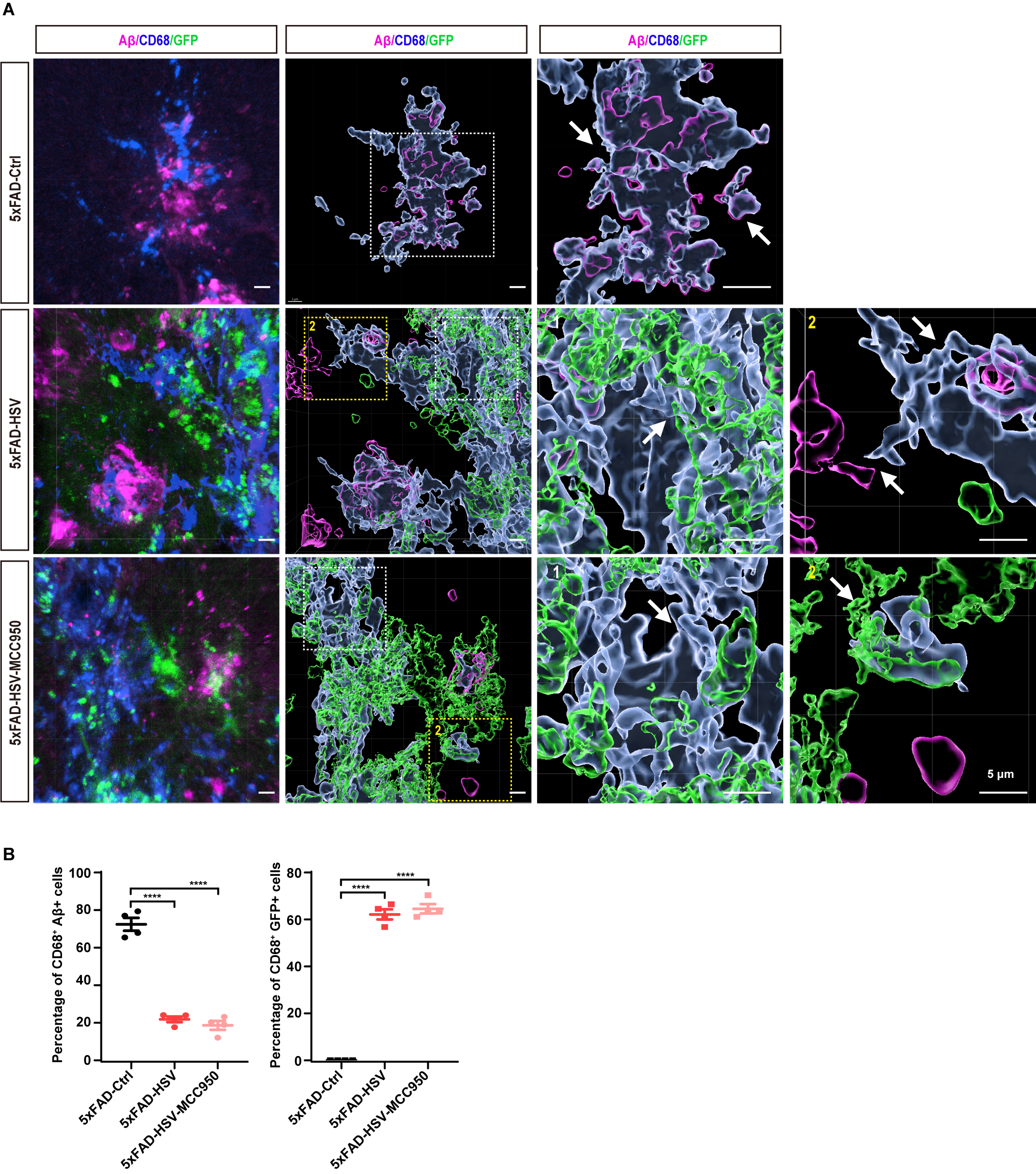

Supplement: Supplementary file 10 — Additional file 10: Figure S10. MCC950 treatment does not affect the phagocytic preference of microglia following HSV-1 infection in 5xFAD mice. (A) Representative images of phagocytic microglia (CD68, blue) co-stained with Aβ plaques (magenta) in the hippocampus of 5xFAD mice treated with MCC950 for 21 days following HSV-1 infection. Three-dimensional reconstructed enlarged images of dashed-white or dashed-yellow frames show engulfed Aβ plaques or GFP-positive cells in the microglia. Scale bars, 5 μm. (B) Quantification of engulfed Aβ plaques or GFP-positive cells in (A). (n = 4 mice per group). Data are presented as means ± SEM. ****p ≤ 0.0001. [file 12974_2024_3166_MOESM10_ESM.tif]
